# Objectively measuring the association between the built environment and physical activity: a systematic review and reporting framework

**DOI:** 10.1186/s12966-022-01352-7

**Published:** 2022-09-14

**Authors:** Francesca L. Pontin, Victoria L. Jenneson, Michelle A. Morris, Graham P. Clarke, Nik M. Lomax

**Affiliations:** 1 Leeds Institute for Data Analytics, University of Leeds, Clarendon Way, Leeds, LS2 9JT UK; 2 School of Geography, University of Leeds, Leeds, LS2 9JT UK

**Keywords:** Physical activity, Built environment, Accelerometer, Global positioning systems, Geographic information systems

## Abstract

**Background:**

Objective measures of built environment and physical activity provide the opportunity to directly compare their relationship across different populations and spatial contexts. This systematic review synthesises the current body of knowledge and knowledge gaps around the impact of objectively measured built environment metrics on physical activity levels in adults (≥ 18 years). Additionally, this review aims to address the need for improved quality of methodological reporting to evaluate studies and improve inter-study comparability though the creation of a reporting framework.

**Methods:**

A systematic search of the literature was conducted following the PRISMA guidelines. After abstract and full-text screening, 94 studies were included in the final review. Results were synthesised using an association matrix to show overall association between built environment and physical activity variables. Finally, the new PERFORM (’Physical and Environmental Reporting Framework for Objectively Recorded Measures’) checklist was created and applied to the included studies rating them on their reporting quality across four key areas: study design and characteristics, built environment exposures, physical activity metrics, and the association between built environment and physical activity.

**Results:**

Studies came from 21 countries and ranged from two days to six years in duration. Accelerometers and using geographic information system (GIS) to define the spatial extent of exposure around a pre-defined geocoded location were the most popular tools to capture physical activity and built environment respectively. Ethnicity and socio-economic status of participants were generally poorly reported. Moderate-to-vigorous physical activity (MVPA) was the most common metric of physical activity used followed by walking. Commonly investigated elements of the built environment included walkability, access to parks and green space. Areas where there was a strong body of evidence for a positive or negative association between the built environment and physical activity were identified. The new PERFORM checklist was devised and poorly reported areas identified, included poor reporting of built environment data sources and poor justification of method choice.

**Conclusions:**

This systematic review highlights key gaps in studies objectively measuring the built environment and physical activity both in terms of the breadth and quality of reporting. Broadening the variety measures of the built environment and physical activity across different demographic groups and spatial areas will grow the body and quality of evidence around built environment effect on activity behaviour. Whilst following the PERFORM reporting guidance will ensure the high quality, reproducibility, and comparability of future research.

**Supplementary Information:**

The online version contains supplementary material available at 10.1186/s12966-022-01352-7.

## Background

Physical inactivity has a hugely detrimental effect on health [1–3] and is recognised by the World Health Organisation (WHO) as the fourth leading cause of global mortality, behind high blood pressure, tobacco use and high blood glucose [4]. Through their Global Action Plan on Physical Activity (GAPPA), the WHO intends to address this global health issue, aiming for a 15% reduction in the prevalence of global physical inactivity in adults and adolescents by 2030 [5]. To achieve this target, cross-disciplinary approaches are required to encourage activity in different populations.

One modifiable factor, with an established link to individual level physical activity, is the environment in which people live and work. Commonly referred to as the built environment, this is the part of the physical environment that is constructed by human activity [6]. Built environment exposure can be determined by defining a geometric area of exposure around either a geocoded location, such as home address, or around global positioning system (GPS) data points. Often measures of built environment focus on land use mix, street connectivity, accessibility, and density measures [6]. However, the built environment is not limited to these features alone. Definitions also encompass other environmental features for instance safety, aesthetics, access to green space and transport provisions [7, 8]. These components can be both a significant enabler of, or barrier to, being physically active. Hence, one of the four main objectives for achieving the GAPPA is to create active environments [5].

Objective measures of both physical activity and the built environment, or other environmental features, utilising accelerometery and geographic information system (GIS) or GPS methodologies are becoming ever more prevalent in research, with continual technological improvements making these technologies increasingly cost-effective [9]. Objective measures are advantageous in that they provide opportunity for direct comparison. For example, accelerometer counts can be directly compared across studies if the same device brand and algorithm is used. Moreover, objective measures are not limited by the reporting and recall biases associated with self-reported measures of physical activity or environmental exposure, such as inaccurate recall of activity location, duration, or intensity. However, utilising objective measures can limit potential sample size and study duration due to associated costs of providing study participants with trackers and participant burden. Increasingly, objective spatial and accelerometery data from smartphones are being used [10–12], which may avert some of the cost and time limitations of the more traditional pairing of GPS and accelerometer devices. Nevertheless, despite the opportunity presented by using objective methods for direct metric comparability, there is currently limited inter-study comparability, thus preventing meta-analysis [6, 13, 14].

Whilst we know the environment in which we live and interact plays an important role in our opportunity to be physically active, studies investigating the strength and direction of even these objectively measured relationships regularly find conflicting outcomes [15, 16]. Due to the plethora of interacting elements that comprise the built environment, alongside the need to make subjective data handling decisions, concluding which elements are encouraging physical activity is challenging.

Moreover, it is difficult to determine whether an increase in physical activity is a universal outcome of a built environment feature or is in fact individual, neighbourhood, area, or country specific [17]. This is particularly due to the range of factors, for example socio-economic status and cultural and political environments that modify the built environment physical activity relationship at a micro and macro level [18, 19]. The trans-disciplined nature of studies considering the built environment, which span disciplines from transport planning, and geography to, health and sports science, results in different approaches, methodologies, and primary study foci [20]. Consequently, different reporting practices and standards have emerged, causing problems for study comparability [14], and subsequent difficulty in concluding the level of influence that built environment has on physical activity.

The International Physical Activity and the Environment Network (IPEN) have aimed to utilise the comparability and transferability of objective methods to address some of these comparability issues. They have designed a cross-national study using the same objective measures of built environment and physical activity across 14 nations. However, only a handful of cross-study comparisons utilising objective data for both physical activity and the built environment have been published by the IPEN group [21–23]. Moreover, whilst the IPEN study design aims to increase the body of evidence regarding the association between built environment and physical activity, through a unified cross-country study design, the scope of physical activity and built environment metrics used is limited to those available in all countries. Hence, there remains a need to be able to evaluate and synthesise the wide-ranging investigations into varied aspects of built environment and physical activity [22].

Systematic reviews present a key method to keep record of, synthesise and evaluate the ever growing and complex body of knowledge in this subject area [24]. Systematic reviews to date have either focused on studies using self-reported or mixed methods of collecting both built environment and physical activity measures [13, 25–27], or have focused on specific study designs to evaluate causality [27, 28]. McCrorie et al. (2014) have systematically reviewed studies utilising objective measures of both physical activity and built environment but only for child participants and no such review has been conducted in an adult population [9]. Within the field of food environment research Wilkins et al. (2017) have devised a framework (GeoFERN) for reporting studies addressing retail food environments [29].They report data sources, methods for data extraction, classification, geocoding, and definitions of the retail food environment, to evaluate the quality of methodological reporting [29]. The methodologies utilised in both food and built environment research hold many similarities, yet, despite recognition of disparities in reporting and calls for increased quality of methodological reporting [6, 13], no such reporting framework or guidance has been devised for studies investigating the physical activity-built environment. Broader, spatial epidemiology reporting frameworks also exist, however whilst they capture many relevant reporting elements, they are not domain specific [30].

In order to identify the aforementioned inconsistencies and reporting gaps in current studies, and to inform policy at a local, national, or global level, a greater comparability of studies is required. Thus, this paper has two aims. The first aim of this paper is to synthesise the current comprehensive body of literature around the associations of objectively built environment variables with physical activity levels and to define knowledge gaps. The second aim is to devise a Physical and Environmental Reporting Framework for Objectively Recorded Measures (PERFORM) for the evaluation of current studies and to improve interstudy comparability of future work, increasing the robustness of conclusions drawn about the physical activity-built environment relationship.

## Methods

### Protocol and registration details

The review protocol was completed following the PRISMA guidelines [31]. A copy of the protocol is published on the PROSPERO International prospective register of systematic reviews [32] (published on 22/06/18 registration number: CRD42018087274).

### Eligibility criteria

To be eligible for inclusion, a peer-reviewed study had to investigate the relationship between an objective measure of the built environment (e.g., GIS derived exposure around a geocoded location or GPS points) and an objective measure of physical activity (E.g., Accelerometer, Pedometer). As many different activities can contribute to meeting physical activity guidelines, no limitation was put on the type of activity objectively recorded [33]. All study designs were permitted for inclusion. However due to the resources available to the reviewers, non-English language studies were excluded from the final review. In order to compare similar built environments, with comparable levels of infrastructural development, studies must have taken place in an Organisation for Economic Co-operation and Development (OECD) country [34, 35]. Similarly, studies focused only on the workplace environment were not included as workplace studies tend to focus on indoor environments or are company specific interventions that apply to a small subset of the population [36].

Study participants must be adults (≥ 18 years of age) for inclusion in the review with no upper age limit. Studies were only included if participants did not suffer from a disability or long-term health condition as these groups face greater barriers to physical activity than the general population and therefore warrant a separate review [37–39]. High BMI (Body mass Index) of participants, i.e., participants being overweight or obese, was not considered a limiting health criteria as long as they suffered from no other long-term health conditions. There is evidence that women’s physical activity behaviours temporarily change during pregnancy, for example decreasing the intensity of exercise [40]. Therefore, studies looking at pregnancy and the built environment are not directly comparable to the general population and are excluded from the review. If studies investigated non-eligible participant characteristics, but included participants as a control or subgroup, these data were included in the review.

### Information sources

Due to the multi-disciplinarity of the research area, a wide-ranging literature search of 14 electronic databases was conducted, reported in Additional file A. Search terms were split into four categories: (1) Physical activity e.g., movement, walking; (2) Built environment e.g., green space; walkability; (3) Physical activity measurement method e.g., accelerometer, fitness tracker; (4) Build environment measurement methods.

e.g., GIS, GPS. These categories were designed to capture both the elements of interest and the respective methods by which each can be objectively measured. Both key word searches and MeSH medical subject headings, where appropriate for the database, were used and the strategy was reviewed by an information specialist. The search was conducted on the 26/03/2020. An example search conducted in MEDLINER of these as illustrated in Table 1.

**Table 1:** Search Strategy for OVID Databases (MEDLINER (1996- search date)

Search Query
1. exp *locomotion/ or exp *physical fitness/ or leisure activities/ or exp *recreation/ or exp movement/ or exp walking/ or exp physical activity/ or exp physical fitness/ or exp *motor activity/ or exp *physical exertion/ or exp *Sedentary Lifestyle/ or exp *exercise/ or exp sport/
2. (”physically active” or”physical activity” or walk* or cycle or bicycle or cycling or sport or sports or exercis* or distance or”active living” or”active transport*” or movement or steps or inactivity).tw
3. 1 or 2
4. exp Fitness Trackers/ or exp Accelerometery/ or exp Smartphone
5. ((fitness adj2 monitor*) or (fitness adj2 track*) or (fitness adj2 wearable) or (fitness adj2 electronic) or (activity adj2 monitor*) or (activity adj2 track*) or (activity adj2 wearable) or (activity adj2 electronic) or (smart adj2 phone) or acceleromet* or pedometer).tw
6. 4 or 5
7. exp *rural health/ or exp *suburban health/ or exp *urban health/ or exp *population/ or exp *Residence Characteristics/ or exp *Environment Design/ or exp *social planning/ or exp *”Sports and Recreational Facilities”/ or exp *Geography, Medical/
8. (”built environment” or geospatial* or”environmental health” or”healthy environment” or (environmental adj1 design) or (urban adj2 health) or walkable or neighbourhood? or greenspace or”green space” or bikeab* or pedestrian).tw
9. 7 or 8
10. exp Geographic Information Systems/
11. (GIS or”geographic information system” or GPS or”global positioning system”).tw
12. 10 or 11
13. 3 and 6 and 9 and 12
14. remove duplicates from 13

### Study screening and data collection process

All titles and abstracts were imported into the online COVIDENCE tool and double screened by F.P and V.J for relevance using the inclusion and exclusion criteria, using COVIDENCE to help identify conflicts and duplicated abstracts [41]. All conflicts were discussed by F.P and V.J and any further disagreement was settled by the third reviewer M.M. The remaining eligible studies then underwent full-text review using the same double review process by F.P and V.J as before. If studies were not fully available online authors were contacted to gain access.

Data Extraction of the studies was completed utilising a predetermined data extraction form developed by the lead author using guidance outlined by Higgins et al. [42]. A sample of included texts were used to pilot the form to ensure it captured all the information required to meet the aims of the review. Data Extraction was conducted by F.P and a sample (*N* = 5) verified by V.J. The completed data extraction form is available in Additional file B. In brief, information extracted included study design, location and duration, information regarding participant recruitment as well as any recorded socio-economic and demographic covariates investigated alongside the built environment and physical activity association. Detailed definitions, measurement methodologies and baseline results were collected for both the built environment exposures and physical activity outcomes reported in the studies. For example, the built environment variable of interest was recorded e.g., green space, alongside the geographic method used, e.g., a 400 m buffer around the home location, paired with the physical activity outcome of interest, e.g., Moderate to Vigorous Physical Activity (MVPA) and finally the threshold of the physical activity measure e.g., 2200 accelerometer counts per minute (CPM). In addition, a null, statistically significant positive association or significant negative quantifiable measure of the association between the built environment and physical activity was recorded. Many papers utilised subsets of data from larger studies. In such cases the original published study was primarily used, and the subsequent papers compiled to aid comprehensive completion of the data extraction form.

### Data synthesis

Due to the wide range of methodologies employed across the included studies, a comprehensive meta-analysis of the resulting metrics was not possible. Therefore, a narrative synthesis was conducted and variations in study population, duration, covariate controls and the definitions of the exposures and outcomes were summarised. In addition, the association between built environment and physical activity and the methods used to objectively capture them was explored, as outlined below.

#### Quantifying built environment and physical activity association

The aforementioned extracted associations between physical activity and built environment were recorded in an association matrix in order to identify areas of strong or weak association, as well as gaps in the current literature. Overall association between the two measures was calculated using the following formula for each pair of associations: 

$$\mathrm{BE}-\mathrm{PA}\;\mathrm{association}\;=\frac{(i\times-1)+(k\times1)}{i+j+k)}$$

where:

*i* = count of all statistically significant negative BE-PA associations *j* = count of all null BE-PA associations.

*k* = count of all statistically significant positive BE-PA associations.

The total number of associations investigated (*i* + *j* + *k*) was also recorded. Not all studies were suitable for inclusion in the association matrix, for instance studies that did not calculate significance of association(s) or those where the direct (or modified) association(s) between singular built environment and physical activity variables was not possible e.g., Latent profile analysis where the profiles are built from multiple aspects of the built environment.

### Developing the Physical and Environmental Reporting Framework for Objectively

#### Recorded Measures (PERFORM)

To meet the second aim of this research, aiding in the synthesis of the extracted data and increasing comparability of future reporting, a reporting framework was devised—“Physical and Environmental Reporting Framework for Objectively Recorded Measures” (PERFORM). The goal of the reporting framework checklist is to improve inter-study comparability of the objective studies to widen the evidence base and increase potential research opportunities. Often the data collected in these studies could be used to address further research questions, however this is not possible if the methods of data collection and processing are not clearly reported. The reporting framework aims to increase research reproducibility and replicability within the field. For instance, using comparable data collected in two different studies to compare the same built environment exposure in different countries or facilitating study repetition to allow longitudinal analysis.

First, commonly comparable elements of reporting across all studies were characterised into four thematic areas:

1. study design,
2. objective built environment methods and data collection,
3. objective physical activity methods and data collection,
4. method of quantifying the association between physical activity and built environment.

The initial elements were developed by the authorship team and refined iteratively during the data extraction stage. During the data extraction stage of the review, common reporting gaps, and/or missing details were identified within these thematic areas. Thus, the reporting framework directly reflects where there are reporting limitations within the current literature. Key questions and examples were then constructed to encourage clear reporting in these areas. The checklist was also designed to capture the seven domains of the bias included in the ROBINS-I framework: Bias due to confounding, bias in selection of participants into the study, bias in classification of interventions, bias due to deviations from intended interventions, bias due to missing data, bias in measurement of outcomes, bias in selection of the reported result [43]. The checklist points were then refined using a random sample (*N* = 5) of studies included in the review [43]. The checklist will aid in future comparison of studies as a result of replicable reporting practices. The final PERFORM checklist is detailed in Table 2.

**Table 2:**
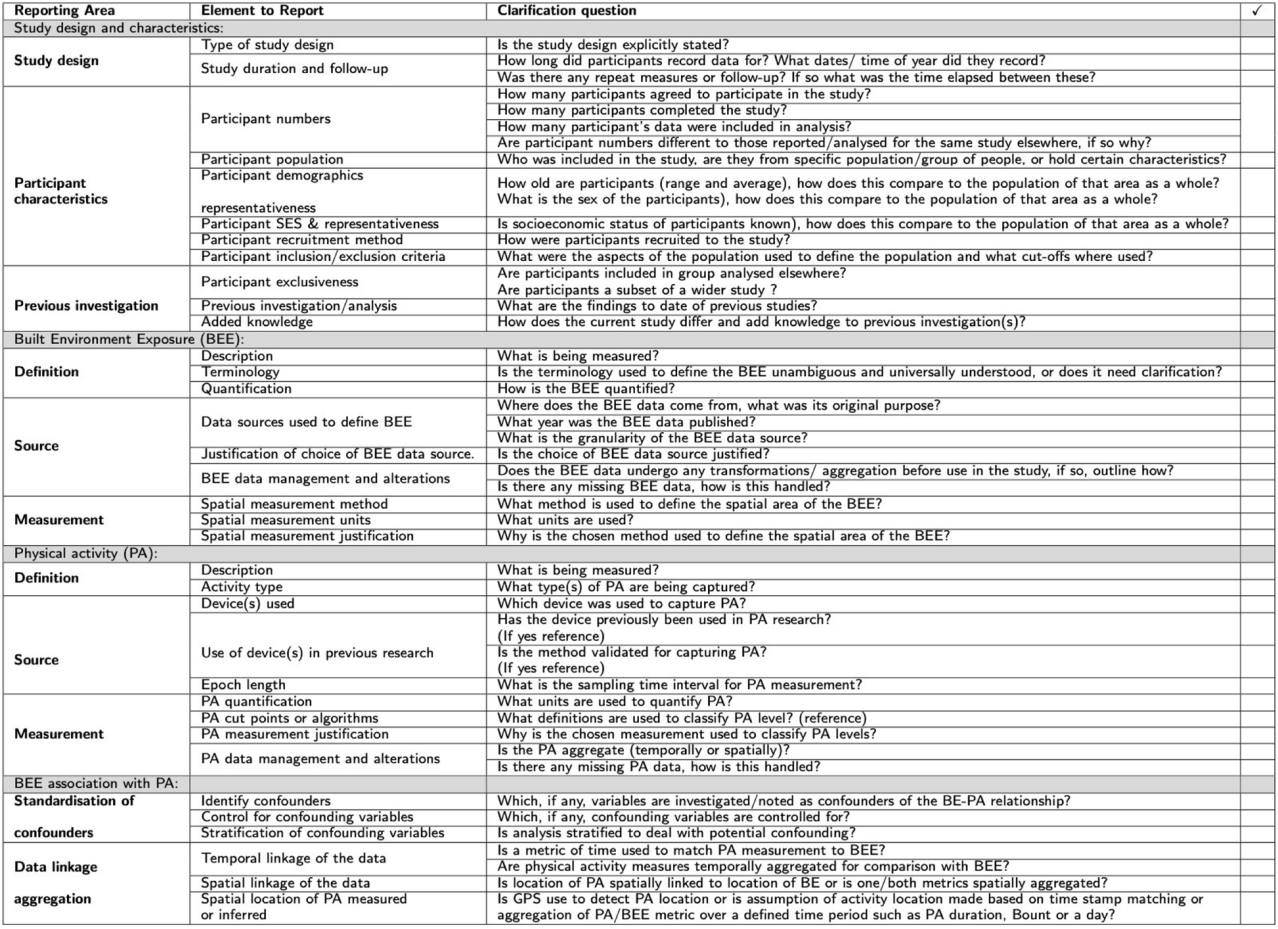
PERFORM: Physical and Environmental Reporting Framework for Objectively Recorded Measures

### Evaluation of study reporting quality using PERFORM

The reporting framework was then utilised to review the quality of studies included with the systematic review and assess risk of bias. Each study was assessed against the reporting criteria and given a score based on whether each criteria of the checklist were reported in the study methodology and results (1.0: reported, 0.5: partially reported, 0: Not reported, NA: criteria not applicable to study design). The proportion of elements reported was then obtained for each paper, to give a PERFORM score out of 100. Additionally, each criteria on the checklist were scored on how well it was reported across all studies.

## Results

### Study selection

**Fig. 1:**
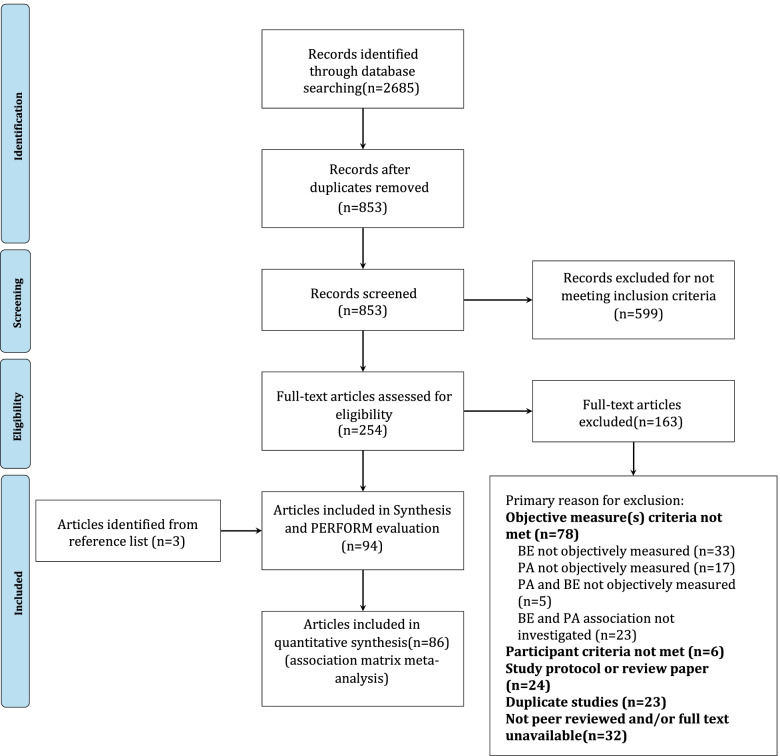
PRISMA flow diagram

The initial literature search identified 2,685 records. Utilising the Convidence tool 1,832 duplicate abstracts were identified and removed before screening (Fig. 1). Abstracts and titles were screened identifying 254 papers as eligible for full-text review and 599 were excluded for not meeting the inclusion criteria. Of the 254 papers that underwent full-text review 163 were excluded, full exclusion reasoning is outlined in Fig. 1. The most common reason for exclusion (78 papers) was the use of non-objective measurements of physical activity and or the built environment (Fig. 1). After full-text screening 91 records were found to be eligible for inclusion, a further three records were identified via reference lists to give a total of 94 included articles.

### Study characteristics

An overview of the study characteristics included in the review can be found in Additional file B. Some articles investigate different aspects of the association between built environment and physical activity in the same participant group and utilising the same study design. In such cases we have grouped these studies for analysis into’projects’, as study concept and design should be homogeneous across all constituent studies and to avoid duplication. Hence the 94 studies meeting inclusion criteria correspond to 64 unique projects. Six of these projects (16 studies) and an additional three summary studies were part of the wider IPEN study. The remaining 55 projects (75 studies) investigated the objective association between built environment and physical activity independent of the IPEN network.

**Fig. 2:**
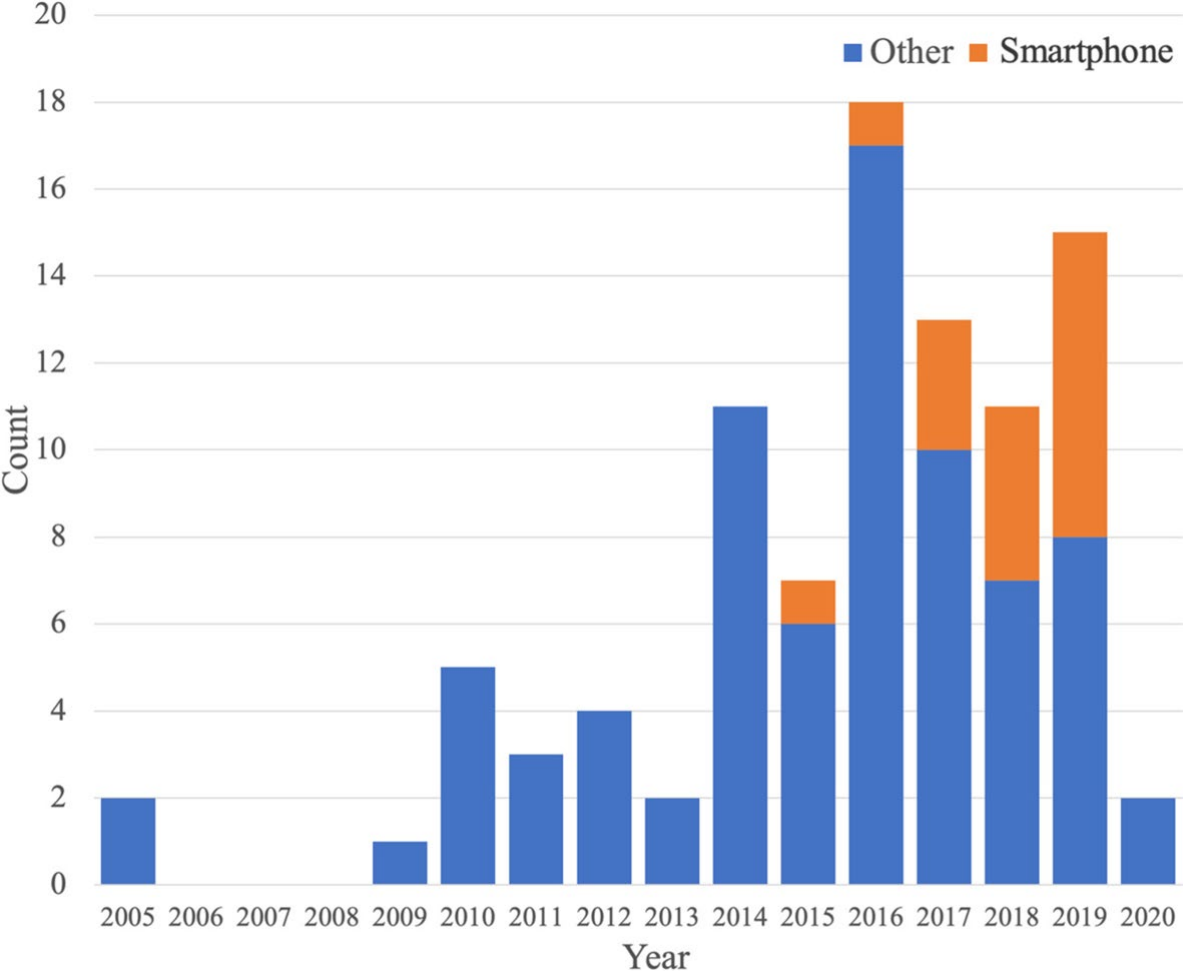
Distribution of article publication date, articles using smartphone data are indicated in orange

Articles were published between 2005 and March 2020 with increasing numbers of publications in recent years, illustrated in Fig. 2. The increase in studies using smartphone data is also demonstrated in the past few years. Mirroring the IPEN network design, cross-sectional studies (71.3%, *N* = 67) were by far the most popular study design utilised to investigate the built environment and physical activity relationship, with a further 13 studies (13.8%) using a case–control study design,

10 (10.6%) followed a quasi-experimental or natural experiment design, two studies (2.1%) were randomised control trials and two (2.1%) were pilot studies that reported a built environment and physical activity association. A seven-day study design for physical activity data collection was most widely used (*N* = 72, 76.6%), though study duration ranged from two days to six years in temporal coverage, as depicted in Additional file B which also indicates if any repeat follow-up measures were taken.

**Fig. 3:**
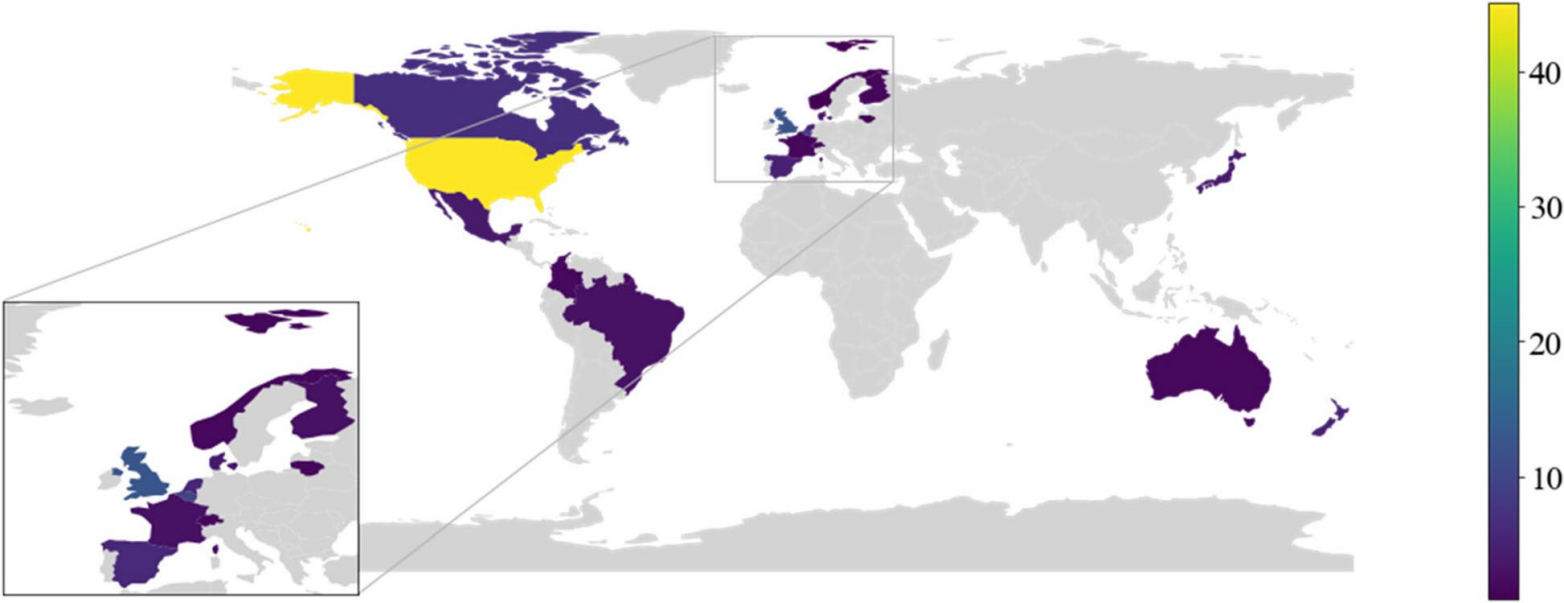
Count of studies from included OECD countries

Figure 3 shows the number of studies in each country, with some studies investigating multiple geographic locations. Studies span 21 OECD countries with 15 unique countries captured by the non-IPEN network studies. The majority of studies (*N* = 56) were conducted in North America and are predominantly USA based investigations (*N* = 45), with European countries (*N* = 47) also forming a large body of the evidence (Fig. 3).

### Participant characteristics

Full participant characteristics are outlined in Additional file B. Participant numbers ranged from 10 to 65,967 unique individuals. 14 studies did not report the gender of participants, the percentage of female participants in the remaining studies ranged from 12.4% to 77.1%, with a median of 54.2%, excluding the four studies looking at only female populations. We grouped studies by age groups to aid interpretation. 43.6% of studies investigated all adults over 18, 33.3% of studies investigated working age populations (18–64 years of age) and 13.8% of studies investigated older adults (65 + years of age). A further three studies (3.2%) specifically looked at student populations (typically under 25 years of age) and 6.4% of studies (*N* = 6) did not report the age of the study participants.

A variety of different population groups were investigated. Participants were often recruited from geographic areas with a specific built environment and/or socioeconomic characteristics. For instance, 21 studies followed the IPEN study design recruiting participants from either a low or high walkable residential area with a low or high socioeconomic status. A further 24 studies investigated participants who live in areas meeting a specific built environment characteristics such as being close to transport or cycling infrastructure developments [43, 44]. Three studies investigated physical activity in ethnic minorities whilst four investigated activity specifically in women. Other groups investigated included trail users (*N* = 2), parents (*N* = 2) and twins (*N* = 1), with 27 studies not limiting the scope of the population included. Recruitment criteria varied widely by study. Common criteria included physical activity criteria such as being able to walk a certain distance unaided (*N* = 35); language proficiency (*N* = 24) and address specifications (*N* = 21).

### Objective data collection

Table 3 summarises the devices and technology used to obtain the objective measures of built environment and physical activity. In order to define the activity space of interest and derive built environment exposure, 24 projects used GIS to define the activity space around a geocoded area. Whilst 26 projects used location data from GPS devices to inform the spatial extent of the activity space area investigated. Accelerometers were the most commonly used device to capture physical activity behaviour with 60 studies solely using accelerometer and 15 studies using accelerometers in conjunction with GPS devices, a further three studies used pedometers.

14 studies used smartphone GPS data to capture the built environment and physical activity behaviour. Of studies utilising smartphones eight used the smartphone as a primary data collection tool and six used secondary smartphone app data; data originally collected for another research purpose [135]. All secondary studies used GPS data to define physical activity, whilst seven primary studies used GPS and two primary studies used the in-built smartphone accelerometer. Secondary data collection led to on average larger sample sizes with up to 13,684 users [133, 134] or 1,105,596 trips [130]. However, larger sample size was often at cost to the level of demographic detail collected on participants with only 50% of secondary studies reporting the age of participants. Moreover, of the secondary smartphone studies that reported gender (33% projects) female participants made up only between.

12.4–19.9% of the included study population.

Built environment data sources were generally poorly reported, as shown in Table 4. With 56 studies not fully referencing the data source used to define the built environment. Of these 56 studies, 18 provided no information at all for the built environment source, often stating GIS was used and even the software but not the underlying data used. A further 17 of these 56 studies non-explicitly mention the data source, for instance that building plan data was used, but not where it was from, with 18 studies mentioning the data provider but not referencing it or stating the year in which it was published. For one study the environment of interest was the home neighbourhood therefore no additional built environment data was required.[96].

**Table 3:** Devices and technology used to provide objective measures of built environment and physical activity

Objective reporting characteristics	Study count(projects)	Papers
Built environment data recording		
GIS used to define spatial extent around reported or inferred geocoded location(s)	37 (24)	[22, 23, 45–54] [55–66] [67–78] [79]
GIS used to define spatial extent around data from a GPS device	41 (26)	[80–91] [92–103] [104–112] [113–120]
GIS used to define spatial extent around GPS data from a smartphone, used as a primary data collection tool	9 (8)	[121–129]
GIS used to define spatial extent around secondary smartphone app GPS data	7 (6)	[10, 44, 130–134]
Physical activity data recording		
Accelerometer	60 (42)	[22, 45–51, 81–84] [23, 52, 53, 55, 85–91] [56–62, 92–95] [64–66, 96–98, 101, 102, 104–106] [67–75, 107, 109] [76–78, 120]
Accelerometer & Pedometer	1 (1)	[80]
Smartphone app: Primary (GPS)	7 (6)	[121–123, 125–128]
Smartphone app used as a primary data collection tool (using phone accelerometer or gyroscope)	2 (2)	[124, 129]
Secondary smartphone app GPS data	7 (6)	[10, 44, 130–134]
Pedometer	2 (2)	[54, 79]
Accelerometer paired with GPS data	15 (5)	[63, 99, 100, 103, 108, 110–114] [115–119]

**Table 4:** Built environment data source reporting

Built environment data source stated	Study count	Papers
No data source info	18	[45, 50, 51, 53, 56, 59, 64, 67, 68, 84, 105, 126] [70, 107, 108, 111, 118, 119]
Source non-explicitly mentioned	17	[22, 66, 69, 80, 86, 87, 91, 97, 99–101, 124] [71, 72, 115–117]
Source stated not referenced	21	[49, 54, 57, 58, 81, 92, 122, 123, 131–133] [73–75, 77–79, 98, 112–114]
Source fully referenced	37	[46–48, 52, 82, 83, 85, 121, 125, 127, 130] [10, 23, 44, 55, 60, 88–90, 128, 134] [61–63, 65, 93–95, 102–104] [76, 106, 109, 110, 120, 129]
NA	1	[96]

### Participant covariates and controls

**Fig. 4:**
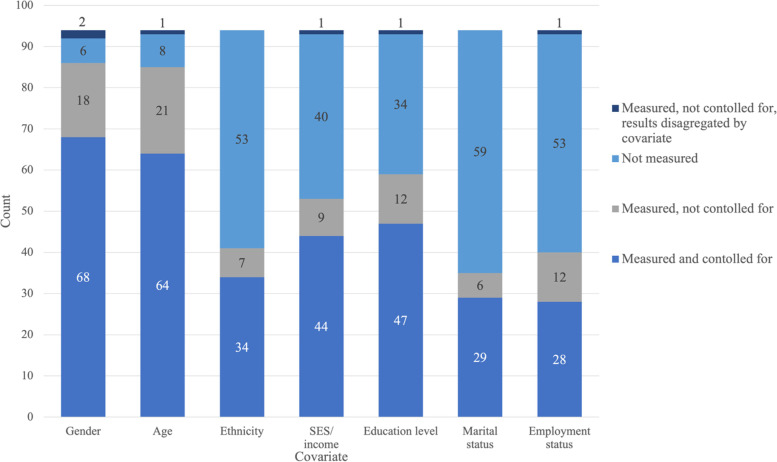
Most commonly reported and controlled for covariates across included studies (*N* = 94)

For each included study the most commonly investigated covariates, outlined in Fig. 4, were reported on whether they were recorded and controlled for in the study design. Generally, age and gender of participants are investigated and controlled for in subsequent analysis. Given that 19 of the 44 studies investigating and controlling for socio-economic status are IPEN studies, designed to capture a broad range of socioeconomic strata, socio-economic status is not particularly well reported in the remaining studies. Ethnicity is also under-reported with only 43.6% of included studies recording a measure of ethnicity. Whilst education, marital status and employment could also be better reported these metrics are less likely to be relevant across all research questions.

### Synthesis of results

#### Quantifying built environment and physical activity association

**Fig. 5:**
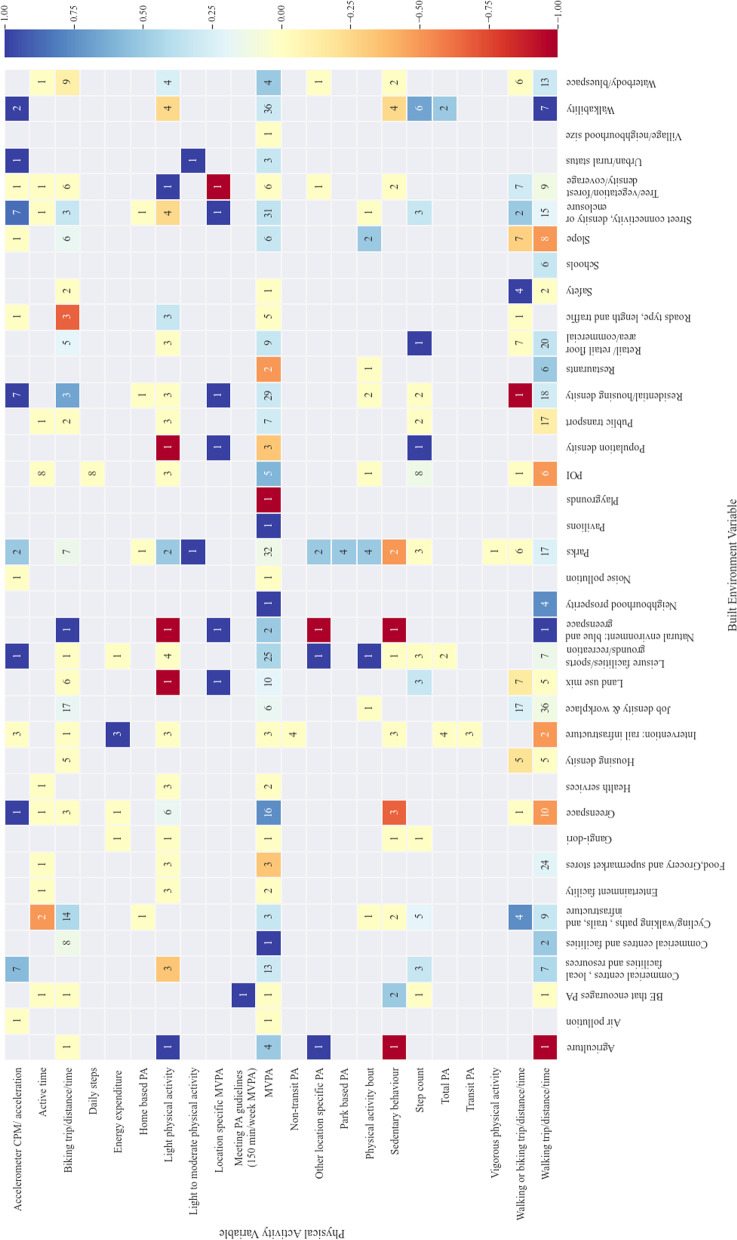
Associations between physical activity and the built environment measures across all included studies (N = 94). The number in the cell indicates number of studies investigating the association and the cell colour indicates the overall association between the metrics across all included studies (1.0 BE promotes PA to -1.0 BE impedes PA)

The range of built environment and physical activity measures used across the included studies are synthesised in the association matrix in Fig. 5. The colour of the cell in the association matrix corresponds to the calculated overall association between the two variables, with the number in the cell indicating the number of instances that association was investigated across the 86 studies that were included in the analysis. The number of built environment and physical activity (BE-PA) associations reported by a single paper ranged from one to 150. Access to and use of parks were the most commonly investigated element of the built environment (84 instances), followed by walkability (61 instances) and measures commonly used to calculate walkability. Residential and housing density (67 instances), job and workplace density (77 instances), street connectivity (69 instances), green space (42 instances), blue space (40 instances) and cycling infrastructure (41 instances) were also popular built environment measures.

As can be seen in Fig. 5 a wide range of physical activity metrics were used.

The most popularly researched physical activity (measured at 284 instances) was MVPA. Walking trips were widely investigated (258 instances) and were also investigated alongside cycling trips a further 76 times, with 104 investigations of cycling as the sole mode of transport. Physical activity definitions also varied widely between studies instigating the same metric. For a full association matrix detailing the methods and cut-points used to define physical activity against built environment metrics see Additional file C.

Methods to define activity space and subsequent built environment exposure varied widely between studies, by spatial method used, size of spatial area and locations(s) investigated. 69 unique spatial methods were used to define built environment exposure. Of those unique methods, 48 were unique definitions using a buffer around home or key locations, varying by buffer size (3 m to a mile) and buffer type with Euclidean buffers accounting for 34 unique methods, (43.4% of investigations in to BE-PA), with street network buffers accounting for the remaining 14 unique methods (18.9% of investigations in to BE-PA). Other popular methods included buffering the daily path area (13 instances), categorising the built environment of GPS point (52 instances), Euclidean distance (17 instances) street network distance (67 instances) and whether the GPS point within the boundary of the area of interest (98 instances).

In terms of physical activity association with the built environment we can start to see where strong evidence of associations have been found (darker blue and red colours) and where that evidence is backed by a larger body of evidence (a larger number in the cell). For instance, in terms of MVPA a strong association is seen with green space (association = 0.75) across 16 instances. Similarly, leisure facilities (25 instances), street connectivity (31 instances) and walkability (36 instances) are also well investigated associations with MVPA and additionally show a physical activity promoting association of 0.44, 0.35 and 0.31 respectively. However, this association is not as strong as that for green space. Parks on the other hand are investigated in association with MVPA on 32 instances however only a moderate positive association is observed (association = 0.13). By comparison, walking trips (17 instances) have a stronger association with parks. However, when it comes to green space (10 instances) walking trips have a strong negative association of -0.5. Poorly investigated aspects of the built environment can be identified for instance safety (9 instances) and air pollution (2 instances). Where there are single studies or no studies investigating an association, these may highlight areas for further investigation, for instance land-use mix and light physical activity (1 instance).

#### PERFORM components

**Fig. 6:**
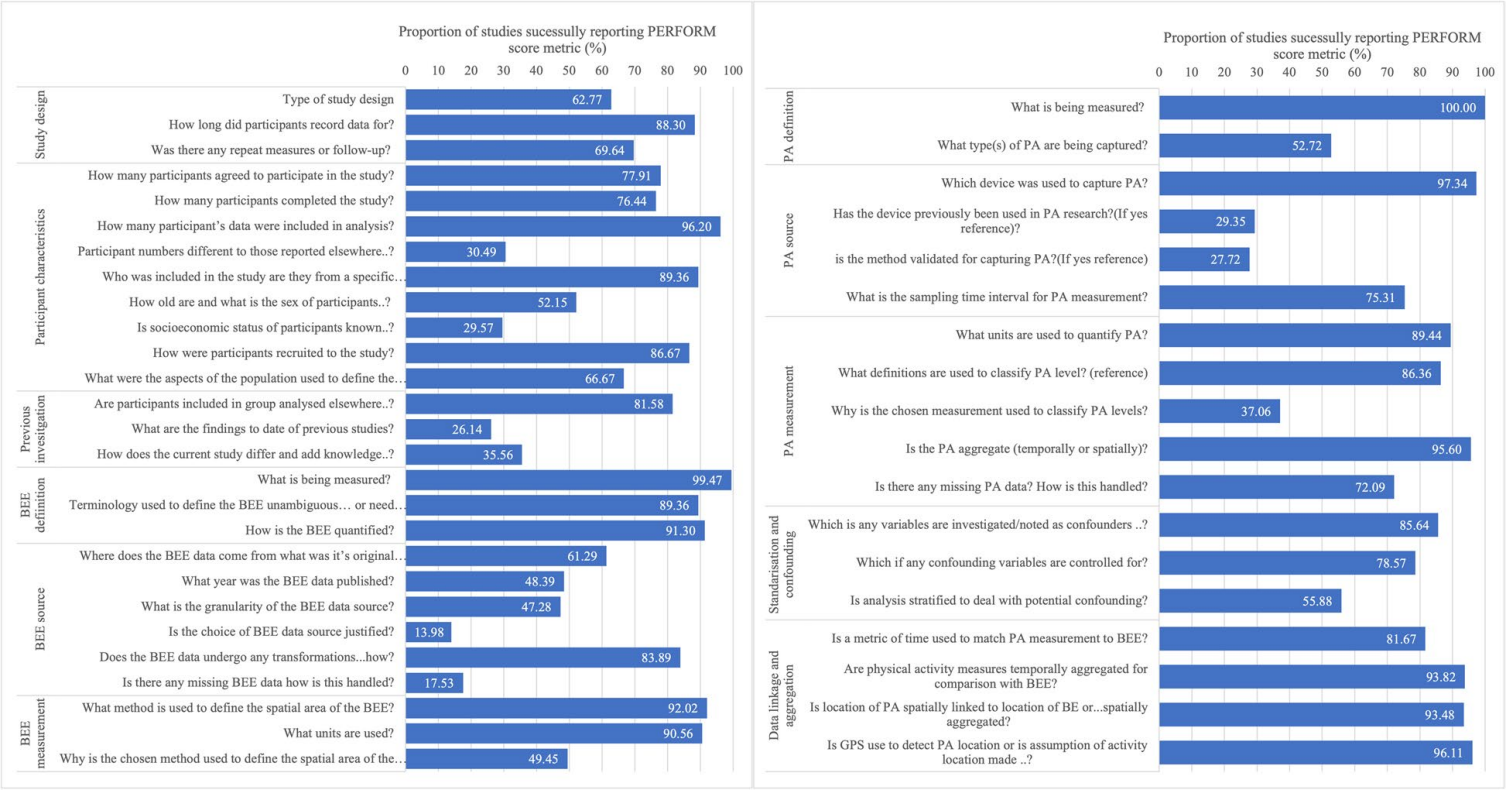
Proportion of studies meeting each PERFORM checklist criteria. (BEE: Built Environment Exposure, PA: Physical activity)

The finalised reporting framework is outlined in Table 2. Figure 6 depicts the proportion of the 94 included studies that report each item, where relevant, in the PERFORM checklist. Reporting ranged from only 14.0% of studies justifying the choice of built environment data source to 100% of studies defining the physical activity measure of interest (Fig. 6).

With regard to study design, participant recording duration was fairly well reported. However, if undertaken, the time duration between initial and follow-up measure was poorly reported, with often only an approximate follow-up lag time given (Fig. 6). As previously mentioned, cross-sectional study designs were by far the most prevalent study design. Nonetheless, reporting of the study deign was mixed or often only included within the limitations to evidence that a causal relationship could not be established (Fig. 6). Participant characteristics were also fairly well reported. However, recruitment criteria and variation in study numbers between studies using the same data were less well reported (Fig. 6). Moreover, whilst the included population characteristics may be summarised and controlled for, a limited number of studies attempted to measure the representativeness of the included participants of the general population (Fig. 6). Although most studies using data from a wider project do reference the said project, often the results of previous studies (26.1% of studies) using these data and the’value add’ (35.6% of studies) of the investigation are not well reported within the studies (Fig. 6).

Generally, the terminology used to define and quantify the built environment is well described. However, whilst most studies report the methods to define the spatial extent of the built environment investigated, this is not universal, just under half the studies justify why that measurement is used. Similarly, when it comes to the underlying data used to characterise the built environment, this is on the whole poorly reported with 61.3% of studies reporting where the built environment data comes from. However, this is reduced when looking at reporting of the original data purpose. Lack of referencing of the source data lead to the low reporting of year of publication (48.4% of studies) granularity of the data source (47.3% of studies). If data is referenced very few studies, then go on to justify why that data source was chosen (14.0% of studies) or if there was any missing data and how this was handled (17.5% of studies). Conversely missing physical activity data and physical activity metric justification are better reported, with 72.1% and 37.1% of studies reporting these PERFORM checklist items respectively, though reporting practices could be improved. The device used to capture physical activity is well reported, nonetheless reference to whether the device has been used previously (29.5% of studies) and whether or not it is a validated method, for the population and setting being investigated (27.7% of studies), need further reporting consideration. Data linkage and aggregation scored fairly well, but often the level of aggregation was implied from the physical activity measurement used e.g., MVPA per day and not reported explicitly.

### Study reporting quality evaluation using PERFORM

**Fig. 7:**
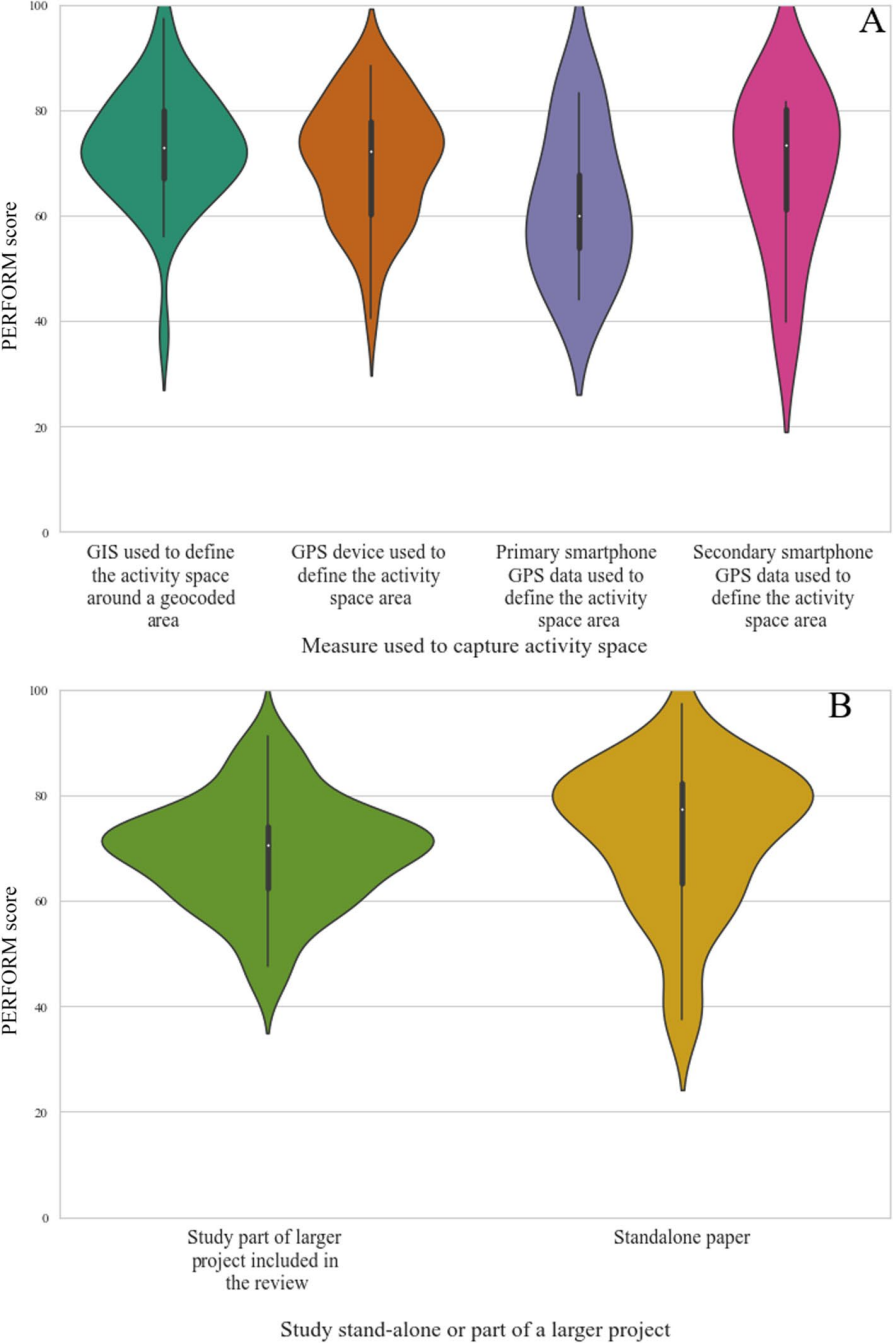
Violin plots depicting the distribution of studies that **A** Capture activity space suing. GIS around a geocoded area, GPS devices, Primary or Secondary smartphone data. **B** Stand-alone studies or are one in a series of papers reporting on the same data

Papers included in the review had a median PERFORM score of 72.2 (IQR:16.5), with scores ranging between 37.5 and 97.4 out of a possible 100. The violin plots in Fig. 7 show the distribution of PERFORM scores and median score by activity space measure (Fig. 7A) and whether the study was part of a wider project (Fig. 7B). Studies that use GIS around a geocoded locations have slightly better reporting practices than those studies using a GPS device to define the activity space. Studies using GPS data from secondary app sources on average report metrics fairly well however, as shown by the large range of scores, this is not universal across all studies using secondary data. Conversely, studies using primary data generally do not score highly using the PERFORM reporting framework. Reporting practices are generally better in stand-alone studies, compared to studies that use the same population as other investigations to answer unique research questions.

## Discussion

### Synthesis of the current body of evidence

The current body of knowledge covering the association between objective measures of the built environment and physical activity is extensive, with 94 studies included in the review. An increase in study numbers in recent years indicates this evidential body is only set to grow. Smartphones were also found to be an increasingly popular method of data collection, with technological advances and reductions in cost making this a more attractive objective method [136]. Study design is important in determining the nature of the conclusions we can draw concerning the built environment influence on physical activity. The majority of the studies included in the review were cross-sectional, which aided cross-country comparison, but prevented conclusions of a causal relationship [76]. Another common experimental design feature was the seven-day recording period for physical activity. Though this has previously been shown to be sufficient to capture habitual activity [137, 138], Bergman et al. have shown that at least 34.8 days of activity were needed to capture MVPA [139]. Moreover Pontin et al. (2021) have shown that intra-individual weekly activity behaviours vary across the year and by season [140].

In terms of population characteristics, a large proportion of included studies were conducted in North America. Caution is therefore needed in applying the findings from these studies to the wider spatial contexts. For instance, one of the most investigated measures, walkability, is based on street connectivity and land use mix. Compared with the USA, European cities have distinctly different designs, for example low levels of street-network sprawl [141], and therefore different walkability scores [142]. Thus, walkability scores will not be directly comparable across cities despite being calculated in the same way and the same increase in walkability in two places may have different impacts on physical activity behaviour.

Investigating population characteristics, we also find women tend to be overrepresented in the included studies, with gender commonly adjusted for, despite evidence that this adjustment modifies the association between built environment metrics and physical activity outcomes. For instance, Tcymbal et al. (2020) find women were more influenced by public transport, safe cycling lanes and housing density, whilst men were more influenced by traditional aspects of walkability such as street network connectivity [143]. Future work therefore would benefit from sub setting built environment and physical activity association analysis by gender.

Evidence from the association matrix showing the strength of associations between built environment and physical activity can be used to guide policy in creating active environments for physical activity [5]. The association matrix also highlights the combination of built environment and physical activity variables are well investigated versus where there are reporting gaps, guiding the design of future research. We therefore recommend consulting the association matrix when looking to design future studies. Moreover, whilst care must be taken to consider potential confounders the matrix provides an indication of the modifiable built environment aspects that could be used to increase activity levels, by activity type. For instance, whilst there is a strong evidence base for MVPA being higher in green space there is a negative association with walking trips. By understanding this detail, we can improve our understanding of how built environment features interact to promote or deter physical activity.

### Recommended future research directions

Reflecting on the strengths and limitations of the current body of evidence we make the following recommendations to guide the direction of future research within this field. First, as suggested by Smith et al. in their review into the implications for causality in the use of activity space measures, a shift away from the favoured cross-sectional study design to natural experiments would strengthen the ability to identify causal relationships within the research field [76]. We suggest revisiting some of the data collected by studies in this review, to identify opportunities for natural experiments and second waves of data collection. For example, re-collecting data to investigate improved pedestrian infrastructure since the original study was conducted. However, to successfully implement natural experiments clear publication of prior analysis and better reporting is required [144]. Second, greater steps need to be taken towards promoting data sharing and publication of meta-data for secondary data analysis to maximise the potential of the existing data [145]. This needs to be undertaken in line with proper consideration of ethics and implementation of data sharing agreements [146].

Fourth, primary barriers to a longer than seven-day study duration are cost and participant burden. Therefore, we encourage the development of smartphones as both a primary and secondary objective data collection tool, due to their low cost, ubiquity in daily life and ability to collect both location and physical activity data [135, 147]. However, secondary smartphone app data are often limited in terms of data on the socio-demographic and other covariates and care need to be taken around missing data [133, 135, 136]. Consequently, research questions need to be carefully considered to ensure these data are suitable or that their use may supplement more traditional study designs. As we demonstrated the quality of reporting in studies utilising these data was highly varied and, in some cases, very poor. Therefore, care must be taken to ensure these studies are reported to the same standard and to enable comparability to more traditional study designs, particularly as these data may be adopted by those coming from a background in data science and not embodied in the literature.

### PERFORM: Recommendations to improve reporting practice

The PERFORM reporting framework identifies four key reporting areas: study design and characteristics, built environment exposures, physical activity metrics, and the association between built environment and physical activity. The reporting framework ensures sufficient detail to replicate the objective measures, both physical activity and built environment, alongside reporting of participant characteristics and uniqueness were identified as the poorest areas of reporting and serves as a checklist to make this reporting easier for researchers. Reporting of results that have been previously published using the same participant populations also need to be better sign-posted and the ‘value add’ reported to ensure the full picture of the interaction between built environment and physical activity behaviour is understood in one population group before we start to compare cross-study populations.

In their 2009 ‘state of the science’ review into measuring the built environment for physical activity, Brownson et al. (2009) propose technical improvements are driving wider measures of the built environment, recommending them to be studied then streamlined into ‘second-generation measures’ [14]. As illustrated in this review, with the ever-decreasing cost of objective measurement technology and the increase in data availability the breadth of these ‘second-generation’ built environment measures is ever expanding. Moreover, the increasing use of smartphones, with their advantages in capturing large populations over wider spatial contexts, could be considered a new third-generation measure. Though the volume of potential built environment measures is not necessarily problematic we need to ensure we understand current evidenced associations. PERFORM is therefore timely in that it can help streamline the reporting of these measures, rather than the measures themselves, to better understand the state of the science today and as we move towards third-generation measures.

In the move towards reproducible research, such a reporting framework is apt [148]. The technical elements around the collection of both accelerometery and built environment data are some of the easiest aspects to report but will go the furthest to ensure replicability by other researchers in different spatial contexts or in the same spatial context after changes to the built environment. PERFORM guidance includes technical aspects such as reporting of accelerometery measurement frequency, mean wear time and non-wear time definitions, alongside how these are combined with GPS frequency measures. For studies investigating built environment using purely GIS to define the spatial extent of the built environment around a geocoded location multiple standardised buffer sizes are suggested to increase study inter-comparability. Moreover, detailed definitions of the physical activity measures and cut-points or algorithms designed to quantify intensity are needed to ensure like-for-like comparison. Justification of data sources used to quantify the built environment exposure is important. Greater transparency is needed regarding how the data are obtained and why these data were chosen. In many instances the data utilised may be the only source available to the researcher. In such cases this should be stated as the reason for use. Completeness, recentness, and granularity of the data are all potential limitations to the study design and exclusion of this detail limits study reproducibility.

### Future implementation of PERFORM

Future researchers should look to employ the PERFORM checklist to aid in the study design and reporting stages of the research. This field is ever evolving so whilst the PERFORM framework will aid in the reporting of the current literature it needs to be regularly reviewed and refined to ensure reproducibility as methods develop and advance. The PERFORM checklist can also be employed, as it is in this review, to identify reporting quality of objective studies in other populations and settings, for instance children or low- and middle-income countries. As objective measuring technologies develop, we recommend an update of this systematic review, reporting against the elements of the PERFORM framework, to ensure ongoing reproducibility within the field.

### Strengths and limitations of the review and framework

This is the first review to look at solely objective measures of physical activity and the built environment. It provides a holistic overview of the current evidence base covering the associations between built environment and physical activity and it highlights where the evidence is lacking or poorly reported. The developed reporting framework, though common in other disciplines, is the first of its nature, designed to improve reporting practices in this increasingly cross-disciplined field.

This paper answers the calls of previous ‘review of reviews’ to provide more information on environmental and physical activity measurement modes to inform future research and practice [149, 150]. Previous reviews have also highlighted the need for better reporting practices [6]. The introduction of a framework will improve comparability of studies by introducing a consistent reporting structure. Therefore, is highly advantageous in forming conclusions for policy and practice. Nonetheless, this review has some limitations. Though this review focuses on objective measures of built environment and physical activity we acknowledge the value of self-reported and perceived measures of built environment and physical activity, with associations between perceived variables often different to those observed via objective measures [151]. That said, many aspects of the reporting framework are relevant and could be adapted to self-reported measures.

In this review we synthesise the association between objective built environment and physical activity by calculating an overall association between the variables in all studies. This metric whilst useful for providing a broad overview into the current body of evidence, uses a crude binary metric of association and does not account for the different statistical methods employed by the individual studies, the covariates controlled for or the spatial context of the study. Thus, these measures of association should be treated as a general outline of the current evidence and be utilised alongside the constituent studies to inform conclusions to overall built environment and physical activity associations.

Though only studies investigating OECD countries were included within this review, to increase inter-study comparability potential, different countries have intrinsically different built environment elements such as the aforementioned street network layout. Caution therefore must be taken when applying evidence and even methods from countries, such as the USA where there is a strong evidence base, to other countries. Other methodological approaches or built environment aspect may prove more suitable in these other contexts, nonetheless the PERFORM reporting framework can still be used for studies employing objective measures in on OECD countries. Therefore, whilst the current body of literature may guide future study design aspects taken of the built environment noted in local qualitative studies may better help quantify the association between built environment and physical activity.

## Conclusions

This review synthesises the current body of knowledge covering the association between objective measures of the built environment and physical activity. With the association matrix providing a valuable reference to future researchers as to areas of strong evidence of environmental association with activity behaviour, such as the association of MVPA with greenspace whilst also highlighting investigatory gaps, strengths and limitations or the current body of evidence. For instance, poor reporting of socio-economic and ethnicity of participants which may confound the relationship between built environment and activity behaviour. The PERFORM reporting framework developed from review of the current body of evidence will improve inter-study comparability of future work and reporting practices. With an increasing volume of data capturing the association between the built environment and physical activity and the development and implementation of PERFORM is timely to improve reproducibility within this research field.

## Supplementary Information

**Additional file 1.** Databases searched.

**Additional file 2.** Data extraction form.

**Additional file 3.** Associations between physical activity (include cut-points) and the built environment metrics across all included studies (*N*=94). The number in the cell indicates number of studies investigating the association and the cell colour indicates the overall association between the metrics across all included studies (1.0 BE promotes PA to -1.0 BE impedes PA).

## Data Availability

The datasets used and/or analysed during the current study are included in this published article [and its supplementary information files] and available from the corresponding author on reasonable request.

## Abbreviations

BMI: Body mass index
BE: Built environment
BEE: Built environment exposure
CPM: Counts per minute
GAPPA: Global action plan on physical activity
GPS: Global positioning system
IPEN: International physical activity and the environment network
MeSH: Medical subject headings
MVPA: Moderate to vigorous physical activity
OECD: Organisation for economic co-operation and development
PA: Physical A = activity
PERFORM: Physical and environmental reporting framework for objectively recorded measures
PRISMA: Preferred reporting items for systematic reviews and meta-analyses
WHO: World health organisation
