# Supplementary material for: Objectively measuring the association between the built environment and physical activity: a systematic review and reporting framework
Source: Int J Behav Nutr Phys Act. 2022 Sep 14;19:119. doi: 10.1186/s12966-022-01352-7 (PMC9476279; doi:10.1186/s12966-022-01352-7)
Supplement: Supplementary file 1 — Additional file 1. Databases searched. [file 12966_2022_1352_MOESM1_ESM.docx]

Additional file A: Databases searched

Database

Global health (OVID)

MEDLINER (OVID)

Transport Database (OVID)

PyscINFO (OVID)

EMBASE (OVID)

SCOPUS

Web of science

PubMed

CINHAL

SportDiscus

ProQuest

ScienceDirect

CENTRAL

Health Technology Assessment Database
