# Supplementary material for: Objectively measuring the association between the built environment and physical activity: a systematic review and reporting framework
Source: Int J Behav Nutr Phys Act. 2022 Sep 14;19:119. doi: 10.1186/s12966-022-01352-7 (PMC9476279; doi:10.1186/s12966-022-01352-7)
Supplement: Supplementary file 2 — Additional file 2. Data extraction form. [file 12966_2022_1352_MOESM2_ESM.docx]

Additional file B: Data extraction form

|  | | | |  | | | | | | | | |  | | | | | | | | | | | | **participant population** | | | | |  | | | | | | | | | |  | | | | | | | | | | | | | | |  |
| --- | --- | --- | --- | --- | --- | --- | --- | --- | --- | --- | --- | --- | --- | --- | --- | --- | --- | --- | --- | --- | --- | --- | --- | --- | --- | --- | --- | --- | --- | --- | --- | --- | --- | --- | --- | --- | --- | --- | --- | --- | --- | --- | --- | --- | --- | --- | --- | --- | --- | --- | --- | --- | --- | --- | --- |
| **Title** | **Authors** | **Published Year** | **Wider study paper count** | **IPEN Study?** | **Continent(s) (Country/tries))** | | **Study Design** | | **Duration (days)** | | **Total unique sample size** | | **Percentage Female** | | **Age Ranges** | | **Specific**  **Population**  **Groups(s)** | **Recruitment criteria** | **Final Included Participant**  **Demographic**  **Characteristics Described?** | | **Healthy subsection of a**  **wider disease state study** | **Measure of sample**  **representativeness**  **or representative recruitment** | | **PA-BE relationship**    **investigate d in other**  **studies? (is this the first)** | | **Previous PE-BE study**  **series**  **results**  **summarise d in this paper?** | **Method detail**  **reported elsewhere** | | **Smartphone study?** | | | **Spatial data recording** | | **PA data recording** | | **BE data sources stated?** | | **PA data aggregation** | | **Gender as a covariate measured/**  **controlled for?** | | **Age as a covariates**  **measured/ controlled for?** | | **Ethnicity as a covariate**  **measured/**  **controlled for?** | | **SES as a covariate**  **measured/**  **controlled for?** | | **Education level as a**  **covariate**  **measured/**  **controlled for?** | | **Marital status as a covariate**  **measured/**  **controlled for?** | | **Employment t status as**  **a covariate measured/c controlled for?** | |  |  |
| A randomised controlled trial and cost-effectiveness evaluation of  'booster' interventions to sustain increases in physical activity in  middle-aged adults in deprived urban neighbourhoods | Goyder, E.; Hind, D.;  Breckon, J.; Dimairo, M.;  Minton, J.; Everson-Hock, E.; Read, S.; Copeland, R.;  Crank, H.; Horspool, K.;  Humphreys, L.; Hutchison,  A.; Kesterton, S.; Latimer,  N.; Scott, E.; Swaile, P.;  Walters, S. J.; Wood, R.; Collins, K.; Cooper, C. | 2014 | 1 | No | Europe (UK) | | Randomise d-control trial | | 7 days | | 160 | | Not reported | | Working  Population (18-65 years) | | Live in area meeting specific  SES criteria | Meet PA criteria | No participant characteristics described | | No | No metric | | No | | NA | NA | | No | | | GIS inferred | | Accelerometer | | No data source info | | Temporally and Spatially | | Measured and  controlled for | | Measured and  controlled for | | Not measured | | Not measured | | Not measured | | Not measured | | Not measured | |  |  |
| Access to parks and physical activity: an eight-country comparison | Schipperijn, J.; Cerin, E.;  Adams, M. A.; Reis, R.;  Smith, G.; Cain, K.;  Christiansen, L. B.; Dyck, D.  van; Gidlow, C.; Frank, L. D.; Mitas, J.; Pratt, M.; Salvo,  D.; Schofield, G.; Sallis, J. F. | 2017 | 1 | Yes | Europe (Belgium,  Denmark, Czech Republic, UK,),  South America  (Brazil), North  America(Mexico,  USA), Oceania (New Zealand) | | Cross-sectional | | 7 days | | 6181 | | 52.70% | | Working  Population (18-65 years) | | Live in area meeting specific  BE + SES criteria | None | Yes (only final) | | No | No metric | | Yes (this is a  subsequent paper) | | Papers ref  but result not  summarise d | Yes, in referenced  study or protocol | | No | | | GIS inferred | | Accelerometer | | Source nonexplicitly  mentioned | | Temporally and Spatially | | Measured and  controlled for | | Measured and  controlled for | | Not measured | | Measured and  controlled for | | Measured and  controlled for | | Measured and  controlled for | | Measured and  controlled for | |  |  |
| Agent-based modelling of physical activity behavior and  environmental correlations: an introduction and illustration | Zhu, W.; Nedovic-Budic, Z.;  Olshansky, R. B.; Marti, J.;  Gao, Y.; Park, Y.; McAuley, E.; Chodzko-Zajko, W. | 2013 | 1 | No | North America (USA) | | Cross-sectional | | 21 days | | 10 | | 40% | | Older Adults (65+) | | Live in area meeting specific  BE criteria | Meet PA criteria | Yes (only final) | | No | No metric | | No | | NA | NA | | No | | | GPS device +GIS | | Accelerometer + Pedometer | | Source nonexplicitly  mentioned | | Temporally and Spatially | | Measured, not  controlled for | | Measured, not  controlled for | | Not measured | | Not measured | | Measured, not  controlled for | | Not measured | | Not measured | |  |  |
| Assessing the Contribution of  Parks to Physical Activity using GPS and Accelerometry) | Evenson, K. R.; Wen, F.; Hillier, A.; Cohen, D. A. | 2013 | 1 | No | North America (USA) | | Cross-sectional | | 21 days | | 238 | | 53.60% | | All Adults | | Live in area meeting specific  BE criteria | Meet PA criteria  + language criteria + address | Yes (only final) | | No | No metric | | No | | NA | NA | | No | | | GPS device +GIS | | Accelerometer | | Source stated not referenced | | Temporally | | Measured, not  controlled for | | Measured, not  controlled for | | Measured, not  controlled for | | Not measured | | Measured, not  controlled for | | Not measured | | Not measured | |  |  |
| Assessing urban connectivity using volunteered mobile phone GPS  locations | Galpern, Paul; Ladle,  Andrew; Alaniz Uribe, Francisco; Sandalack,  Beverly; Doyle-Baker, Patricia | 2018 | 2 | No | North America (Canada) | | Cross-sectional | | 6 years | | 156 | | Not reported | | Student Population | | NA | None | No participant characteristics described | | No | No metric | | Yes (first) | | NA | NA | | Yes | | | Smartphone GPS:  primary | | Smartphone app: Primary (GPS) | | Source fully references | | Temporally and Spatially | | Not measured | | Not measured | | Not measured | | Not measured | | Not measured | | Not measured | | Not measured | |  |  |
| Measuring the use of green space with urban resource selection  functions: An application using smartphone GPS locations | Ladle, Andrew; Galpern, Paul; Doyle-Baker, Patricia | 2018 | 2 | No | North America (Canada) | | Cross-sectional | | 6 years | | 156 | | Not reported | | Student Population | | NA | None | No participant characteristics described | | No | No metric | | Yes (this is a  subsequent paper) | | Previous paper(s) not  acknowledged | Yes, in referenced  study or protocol | | Yes | | | Smartphone GPS:  primary | | Smartphone app: Primary (GPS) | | Source stated not referenced | | Temporally and Spatially | | Not measured | | Not measured | | Not measured | | Not measured | | Not measured | | Not measured | | Not measured | |  |  |
| Association between daily physical activity and neighborhood  environments | Kondo, K.; Lee, JungSu;  Kawakubo, K.; Kataoka, Y.;  Asami, Y.; Mori, K.;  Umezaki, M.; Yamauchi, T.; Takagi, H.; Sunagawa, H.; Akabayashi, A. | 2009 | 1 | No | Asia (Japan) | | Cross-sectional | | 7 days | | 112 | | 55.10% | | All Adults | | NA | Meet BE criteria | Yes (only final) | | No | Compared to control Population | | No | | NA | NA | | No | | | GIS inferred | | Accelerometer | | Source fully references | | Temporally and Spatially | | Measured and  controlled for | | Measured and  controlled for | | Not measured | | Not measured | | Not measured | | Not measured | | Not measured | |  |  |
| Associations between time spent in green areas and physical  activity among late middle-aged  adults | Dewulf, B.; Neutens, T.;  Dyck, D. van;  Bourdeaudhuij, I. de;  Broekx, S.; Beckx, C.; Weghe, N. van de | 2016 | 1 | No | Europe (Belgium) | | Cross-sectional | | 7 days | | 138 | | 52.20% | | Working  Population (18-65 years) | | NA | Meet PA criteria  + language criteria | Only characteristics pre-applying  inclusion criteria/ subsection described | | No | No metric | | No | | NA | NA | | No | | | GPS device +GIS | | Accelerometer | | Source fully references | | Temporally and Spatially | | Measured, not  controlled for | | Measured, not  controlled for | | Not measured | | Not measured | | Measured, not  controlled  for, results  disaggregate ed by covariate | | Not measured | | Measured, not  controlled  for, results  disaggregated by covariate | |  |  |
| Associations between moderate to-vigorous physical activity and neighbourhood recreational  facilities: The features of the  facilities matter | Lee, K. Y.; Lee, P. H.; Macfarlane, D. | 2014 | 1 | Yes | Asia (Hong Kong) | | Cross-sectional | | 7 days | | 154 | | 58.40% | | Working  Population (18-65 years) | | Live in area meeting specific  BE + SES criteria | None | Yes (only final) | | No | No metric | | No | | NA | NA | | No | | | GIS inferred | | Accelerometer | | Source fully references | | Temporally and Spatially | | Measured and  controlled for | | Measured and  controlled for | | Not measured | | Measured and  controlled for | | Measured and  controlled for | | Measured and  controlled for | | Measured and  controlled for | |  |  |
| Associations of neighborhood walkability with intensity- and  bout-specific physical activity and  sedentary behavior of older adults in Japan. | Amagasa, Shiho; Inoue,  Shigeru; Fukushima,  Noritoshi; Kikuchi, Hiroyuki;  Nakaya, Tomoki; Hanibuchi,  Tomoya; Sallis, James F; Owen, Neville | 2019 | 1 | No | Asia (Japan) | | Cross-sectional | | 7 days | | 450 | | 43.30% | | Older Adults (65+) | | NA | Criteria required, reference to other study | Yes (only final) | | No | Representative recruitment method | | No | | NA | NA | | No | | | GIS inferred | | Accelerometer | | Source fully references | | Temporally and Spatially | | Measured and  controlled for | | Measured and  controlled for | | Not measured | | Not measured | | Measured and  controlled for | | Not measured | | Measured and  controlled for | |  |  |
| Built environment determinants of pedestrians and  Bicyclists route choices on  commute trips: Applying a new  grid-based method for measuring  the built environment along the  route | Sarjala, Satu | 2019 | 1 | No | Europe (Finland) | | Cross-sectional | | NA | | 73 | | 35.60% | | Working  Population (18-65 years) | | Specific workplace | Criteria required, reference to other study | Yes (only final) | | No | No metric | | Yes (this is a  subsequent paper) | | Papers ref  but result not  summarise d | Yes, in referenced  study or protocol | | Yes | | | Smartphone GPS:  primary | | Smartphone app: Primary (GPS) | | Source stated not referenced | | NA | | Measured, not  controlled for | | Measured, not  controlled for | | Not measured | | Not measured | | Not measured | | Not measured | | Not measured | |  |  |
| Characteristics of the built environment in relation to  objectively measured physical  activity among Mexican adults,  2011 | Salvo, D.; Reis, R. S.; Stein, A. D.; Rivera, J.; Martorell,  R.; Pratt, M. | 2014 | 1 | Yes | North America (Mexico) | | Cross-sectional | | 7 days | | 662 | | 51.90% | | Working  Population (18-65 years) | | Live in area meeting specific  BE + SES criteria | Meet health +address | Yes (only final) | | No | Compared to excluded  Population | | No | | NA | NA | | No | | | GIS inferred | | Accelerometer | | Source stated not referenced | | Temporally and Spatially | | Measured and  controlled for | | Measured and  controlled for | | Not measured | | Measured and  controlled for | | Measured and  controlled for | | Measured and  controlled for | | Not measured | |  |  |
| Does new bicycle infrastructure result in new or rerouted  bicyclists? A longitudinal GPS  study in Oslo | Pritchard, Ray; Bucher, Dominik; Fr√∏yen, Yngve | 2019 | 1 | No | Europe (Norway) | | Natural Experiment | | 56 days | | 113 | | Not reported | | All Adults | | Live in area meeting specific  BE criteria | None | Yes (only final) | | No | No metric | | No | | NA | NA | | Yes | | | Smartphone GPS:  primary | | Smartphone app: Primary  (accelerometer/gyroscope) | | Source nonexplicitly  mentioned | | Unclear | | Measured, not  controlled for | | Measured, not  controlled for | | Not measured | | Not measured | | Measured, not  controlled for | | Not measured | | Not measured | |  |  |
| Estimating a Toronto pedestrian route choice model using  smartphone GPS data | Lue Gregory; Miller Eric J | 2019 | 1 | No | North America (Canada) | | Cross-sectional | | 28 days | | 156 | | Not reported | | Working  Population (18-65 years) | | NA | None | No participant characteristics described | | No | No metric | | Yes (this is a  subsequent paper) | | NA | Yes, in referenced  study or protocol | | Yes | | | Smartphone GPS:  primary | | Smartphone app: Primary (GPS) | | Source fully references | | NA | | Measured, not  controlled for | | Measured, not  controlled for | | Not measured | | Measured, not  controlled for | | Not measured | | Not measured | | Measured, not  controlled for | |  |  |
| Estimating bicycle trip volume for  Miami-Dade county from Strava tracking data | Hochmair, H. H.; Bardin, E.; Ahmouda, A. | 2019 | 1 | No | North America (USA) | | Cross-sectional | | 152 days | | 1,105,596 trips | | Not reported | | Not reported | | NA | None | No participant characteristics described | | No | No metric | | No | | NA | NA | | Yes | | | Smartphone GPS:  secondary | | Smartphone app: Secondary (GPS) | | Source fully references | | Temporally and Spatially | | Not measured | | Not measured | | Not measured | | Not measured | | Not measured | | Not measured | | Not measured | |  |  |
| Estimation of pedestrian density and speed on street network using smartphone spatio-temporal data | Shimizu, K.; Nishi, H.; Kishimoto, T. | 2019 | 1 | No | Asia (Japan) | | Cross-sectional | | 365 days | | 5,000 | | Not reported | | Not reported | | NA | None | No participant characteristics described | | No | No metric | | No | | NA | NA | | Yes | | | Smartphone GPS:  secondary | | Smartphone app: Secondary (GPS) | | Source stated not referenced | | Temporally and Spatially | | Not measured | | Not measured | | Not measured | | Not measured | | Not measured | | Not measured | | Not measured | |  |  |
| Exploring socioecological correlates of active living in retirement village residents | Nathan, A.; Wood, L.; GilesCorti, B. | 2014 | 1 | No | | Oceania (Australia) | | Cross-sectional | | 7 days | | 288 | | 68.10% | All Adults | Residential/ retirement home | | None | | Only characteristics pre-applying  inclusion criteria/ subsection described | No | | No metric | | Yes (this is a  subsequent paper) | Papers ref  but result not  summarise d | | Yes, in referenced  study or protocol | | | No | | GIS inferred | | Accelerometer | | No data source info | | Temporally and Spatially | | Measured and  controlled for | | Measured and  controlled for | | Not measured | | Not measured | | Measured, not  controlled for | | Measured, not  controlled for | | Measured, not  controlled for | | |
| A GPS data-based analysis of built environment influences on bicyclist route preferences | Chen Peng; Shen Qing; Childress Suzanne | 2018 | 1 | No | | North America (USA) | | Cross-sectional | | 3.5 years | | 197 | | Not reported | Working  Population (18-65 years) | NA | | None | | Yes (only final) | No | | No metric | | No | NA | | NA | | | Yes | | Smartphone GPS:  primary | | Smartphone app: Primary (GPS) | | No data source info | | NA | | Measured, not  controlled for | | Measured, not  controlled for | | Not measured | | Not measured | | Not measured | | Not measured | | Not measured | | |
| GPS-Based Exposure to Greenness and Walkability and  Accelerometry-Based Physical Activity | James, P.; Hart, J. E.; Hipp,  J. A.; Mitchell, J. A.; Kerr, J.; Hurvitz, P. M.; Glanz, K.; Laden, F. | 2017 | 1 | No | | North America (USA) | | Cross-sectional | | 7 days | | 360 | | 100% | All Adults | Women | | Meet health + PA | | Yes (only final) | No | | No metric | | No | NA | | NA | | | No | | GPS device +GIS | | Accelerometer | | Source fully references | | Spatially | | Measured, not  controlled  for, results  disaggregated by covariate | | Measured and  controlled for | | Measured and  controlled for | | Measured and  controlled for | | Measured and  controlled for | | Not measured | | Measured and  controlled for | | |
| Green streetscape and walking:  Exploring active mobility patterns in dense and compact cities | Vich, Guillem; Marquet,  Oriol; Miralles-Guasch,  Carme | 2019 | 1 | No | | Europe (Spain) | | Cross-sectional | | 7 days | | 127 | | 59.10% | Working  Population (18-65 years) | NA | | None | | Yes (only final) | No | | No metric | | No | NA | | NA | | | Yes | | Smartphone GPS:  primary | | Smartphone GPS:  primary | | Source fully references | | Temporally and Spatially | | Measured and  controlled for | | Measured and  controlled for | | Not measured | | Not measured | | Not measured | | Not measured | | Measured and  controlled for | | |
| How do they do it: working women meeting physical activity  recommendations | Gell, N. M.; Wadsworth, D. D. | 2014 | 1 | No | | North America (USA) | | Case-control | | 7 days | | 103 | | 100% | Working  Population (18-65 years) | Women | | Meet PA criteria  + employment  criteria | | Yes (only final) | No | | Within sample comparison of included subgroups | | No | NA | | NA | | | No | | GPS device +GIS | | Accelerometer | | No data source info | | Temporally | | Measured and  controlled for | | Measured and  controlled for | | Measured and  controlled for | | Not measured | | Measured and  controlled for | | Measured and  controlled for | | Measured and  controlled for | | |
| Individual, social environmental and physical environmental  barriers to achieving 10 000 steps per day among older women | Hall, K. S.; McAuley, E. | 2010 | 1 | No | | North America (USA) | | Cross-sectional | | 7 days | | 128 | | 100% | Older Adults (65+) | Women | | Meet health + ethnicity | | Yes (only final) | No | | No metric | | No | NA | | NA | | | No | | GIS inferred | | Accelerometer | | No data source info | | Temporally and Spatially | | Measured and  controlled for | | Measured, not  controlled for | | Measured, not  controlled for | | Measured, not  controlled for | | Measured, not  controlled for | | Measured, not  controlled for | | Measured, not  controlled for | | |
| Investigating the association between streetscapes and human walking activities using Google  Street View and human trajectory data | Li, Xiaojiang; Santi, Paolo;  Courtney, Theodore K;  Verma, Santosh K; Ratti, Carlo | 2018 | 1 | No | | North America (USA) | | Cross-sectional | | 30 days | | >6,000 | | NA: anonymous app user | Not reported | NA | | None | | No participant characteristics described | No | | No metric | | No | NA | | NA | | | Yes | | Smartphone GPS:  secondary | | Smartphone app: Secondary (GPS) | | Source stated not referenced | | Temporally | | Not measured | | Not measured | | Not measured | | Not measured | | Not measured | | Not measured | | Not measured | | |
| Objectively measured physical activity in population-  representative parent-child pairs: parental modelling matters and is context-specific. | Bringolf-Isler, Bettina;  Schindler, Christian; Kayser,  Bengt; Suggs, L Suzanne;  Probst-Hensch, Nicole; SOPHYA Study Group | 2018 | 1 | No | | Europe (Switzerland) | | Cross-sectional | | 7 days | | 1059 | | 64.80% | Working  Population (18-65 years) | Parents | | None | | Yes (only final) | No | | No metric | | No | NA | | NA | | | No | | GIS inferred | | Accelerometer | | Source fully references | | Temporally and Spatially | | Measured and  controlled for | | Measured, not  controlled for | | Not measured | | Not measured | | Measured, not  controlled for | | Measured, not  controlled for | | Measured, not  controlled for | | |
| Objectively assessed neighbourhood destination  accessibility and physical activity  in adults from 10 countries: An analysis of moderators and perceptions as mediators | Cerin, Ester; Conway, Terry L.; Adams, Marc A.;  Barnett, Anthony; Cain, Kelli L.; Owen, Neville;  Christiansen, Lars B.; van  Dyck, Delfien; Mit√°≈°,  Josef; Sarmiento, Olga L.;  Davey, Rachel C.; Reis,  Rodrigo; Salvo, Deborah; Schofield, Grant; Sallis, James F. | 2018 | 1 | Yes | | Europe (Belgium,  Denmark, Czech Republic, UK,),  South America  (Brazil, Colombia),  North  America(Mexico,  USA), Oceania  (New Zealand),  Asia (Hong Kong) | | Cross-sectional | | 7 days | | 6526 | | 53.70% | Working  Population (18-65 years) | Live in area meeting specific  BE + SES criteria | | Criteria required but not mentioned | | Yes (only final) | No | | No metric | | Yes (this is a  subsequent paper) | Papers ref  but result not  summarise d | | Yes, in referenced  study or protocol | | | No | | GIS inferred | | Accelerometer | | No data source info | | Temporally and Spatially | | Measured and  controlled for | | Measured and  controlled for | | Not measured | | Measured and  controlled for | | Measured and  controlled for | | Measured and  controlled for | | Measured and  controlled for | | |
| Physical activity and the neighborhood environment in a  heavy snowfall area in Japan: The  role of "Gangi-dori" | Kosaka, S.; Umezaki, M.; Ishikawa, M.; Watanabe, C. | 2014 | 1 | No | | Asia (Japan) | | Case-control | | 7 days | | 55 | | 49.10% | All Adults | Live in area meeting specific  BE criteria | | Meet PA criteria | | Yes (only final) | No | | No metric | | No | NA | | NA | | | No | | GPS device +GIS | | Accelerometer | | Source fully references | | No aggregation | | Measured and  controlled for | | Measured and  controlled for | | Not measured | | Not measured | | Not measured | | Measured, not  controlled for | | Measured, not  controlled for | | |
| Physical activity in relation to urban environments in 14 cities worldwide: A cross-sectional study | Sallis, J. F.; Cerin, E.;  Conway, T. L.; Adams, M.  A.; Frank, L. D.; Pratt, M.;  Salvo, D.; Schipperijn, J.;  Smith, G.; Cain, K. L.; Davey, R.; Kerr, J.; Lai, P. C.; Mitas,  J.; Reis, R.; Sarmiento, O.  L.; Schofield, G.; Troelsen, J.; Van Dyck, D.; De  Bourdeaudhuij, I.; Owen, N. | 2016 | 1 | Yes | | Europe (Belgium,  Denmark, Czech  Republic, UK,),  South America  (Brazil, Colombia),  North  America(Mexico,  USA), Oceania  (New Zealand),  Asia (Hong Kong) | | Cross-sectional | | 7 days | | 6822 | | 53.70% | Working  Population (18-65 years) | Live in area meeting specific  BE + SES criteria | | None | | Yes (only final) | No | | Representative recruitment method | | Yes (this is a  subsequent paper) | Papers ref  but result not  summarise d | | Yes, in this study | | | No | | GIS inferred | | Accelerometer | | Source fully references | | Temporally and Spatially | | Measured and  controlled for | | Measured and  controlled for | | Not measured | | Measured and  controlled for | | Measured and  controlled for | | Measured and  controlled for | | Measured and  controlled for | | |
| Portable global positioning units to complement accelerometry based physical activity monitors | Rodriguez, D. A.; Brown, A. L.; Troped, P. J. | 2005 | 1 | No | | North America (USA) | | Pilot Study | | 3 days | | 35 | | 60% | Working  Population (18-65 years) | NA | | Meet health +address | | Yes (only final) | No | | No metric | | No | NA | | NA | | | No | | GPS device +GIS | | Accelerometer | | Source nonexplicitly  mentioned | | Temporally | | Measured and  controlled for | | Measured, not  controlled for | | Not measured | | Not measured | | Not measured | | Not measured | | Not measured | | |
| Public transit generates new physical activity: Evidence from  individual GPS and accelerometer data before and after light rail  construction in a neighborhood of  Salt Lake City, Utah, USA | Miller, Harvey J.; Tribby,  Calvin P.; Brown, Barbara  B.; Smith, Ken R.; Werner,  Carol M.; Wolf, Jean;  Wilson, Laura; Oliveira, Marcelo G. | 2015 | 1 | No | | North America (USA) | | Quasi-  Experiment  al | | 7 days x 2  (baseline and follow-up) | | 536 | | 51% | Not reported | Live in area meeting specific  BE criteria | | Meet PA criteria  + language criteria | | Yes (only final) | No | | Compared to excluded  Population | | Yes (this is a  subsequent paper) | Results  summarise d | | Yes, in this study | | | No | | GPS device +GIS | | Accelerometer | | Source nonexplicitly  mentioned | | Temporally and Spatially | | Measured and  controlled for | | Not measured | | Measured and  controlled for | | Measured and  controlled for | | Measured and  controlled for | | Not measured | | Measured and  controlled for | | |
| Accelerometer and GPS analysis of trail use and associations with  physical activity | Tamura, Kosuke; Wilson,  Jeffrey S; Goldfeld, Keith;  Puett, Robin C; Klenosky,  David B; Harper, William A; Troped, Philip J | 2019 | 2 | No | | North America (USA) | | Cross-sectional | | 4 days | | 148 | | 52.80% | All Adults | Trail users | | Meet BE criteria | | Yes (only final) | No | | No metric | | Yes (this is a  subsequent paper) | Papers ref  but result not  summarise d | | Yes, in referenced study or protocol  +in this study | | | No | | GPS device +GIS | | Accelerometer | | Source fully references | | Temporally | | Measured and  controlled for | | Measured and  controlled for | | Measured and  controlled for | | Not measured | | Measured and  controlled for | | Not measured | | Not measured | | |
| The built environment and location-based physical activity | Troped, P. J.; Wilson, J. S.;  Matthews, C. E.; Cromley, E. K.; Melly, S. J. | 2010 | 2 | No | | North America (USA) | | Cross-sectional | | 4 days | | 148 | | Unclear | All Adults | Trail users | | Meet BE criteria | | Yes (only final) | No | | No metric | | Yes (first) | NA | | NA | | | No | | GPS device +GIS | | Accelerometer | | Source fully references | | Spatially | | Measured and  controlled for | | Measured and  controlled for | | Measured and  controlled for | | Not measured | | Measured, not  controlled for | | Not measured | | Not measured | | |
| The influence of built environment on walkability using geographic information system | Dygryn, J.; Mitas, J.; Stelzer, J. | 2010 | 1 | No | | Europe (Czech Republic) | | Cross-sectional | | 7 days | | 70 | | 41.40% | Working  Population (18-65 years) | NA | | None | | Yes (only final) | No | | No metric | | No | NA | | NA | | | No | | GIS inferred | | Pedometer | | Source stated not referenced | | Temporally and Spatially | | Measured, not  controlled for | | Measured, not  controlled for | | Not measured | | Not measured | | Not measured | | Not measured | | Not measured | | |
| The usefulness of GPS bicycle tracking data for evaluating the impact of infrastructure change on cycling behaviour | Heesch K.C.; Langdon M. | 2016 | 1 | No | | Oceania (Australia) | | Cross-sectional | | NA | | NA | | 19.90% | All Adults | App users | | None | | Yes (only final) | No | | Compared to control Population | | No | NA | | NA | | | Yes | | Smartphone GPS:  secondary | | Smartphone app: Secondary (GPS) | | Source fully references | | Temporally | | Measured, not  controlled for | | Measured, not  controlled for | | Not measured | | Not measured | | Not measured | | Not measured | | Not measured | | |

| Using GPS data to study neighborhood walkability and  physical activity | Rundle, Andrew G.;  Sheehan, Daniel M.; Quinn,  James W.; Bartley,  Katherine; Eisenhower,  Donna; Bader, Michael M.;  Lovasi, Gina S.; Neckerman, Kathryn M. | 2016 | 1 | No | North America (USA) | Cross-sectional | 7 days | 569 | Not reported | Not reported | NA | None | No participant characteristics described | No | Representative recruitment method | No | NA | Yes, in referenced study or protocol  +in this study | No | GPS device +GIS | Accelerometer | Source fully references | Temporally and Spatially | Measured and  controlled for | Measured and  controlled for | Measured and  controlled for | Measured and  controlled for | Measured and  controlled for | Not measured | Measured and  controlled for |
| --- | --- | --- | --- | --- | --- | --- | --- | --- | --- | --- | --- | --- | --- | --- | --- | --- | --- | --- | --- | --- | --- | --- | --- | --- | --- | --- | --- | --- | --- | --- |
| Exploring potential of crowdsourced geographic  information in studies of active  travel and health: Strava data and  cycling behaviour | Sun, Y. | 2017 | 2 | No | Europe (UK) | Cross-sectional | 365 days | 13,684 users | 13.20% | All Adults | App users | None | Yes (only final) | No | No metric | Yes (first) | NA | NA | Yes | Smartphone GPS:  secondary | Smartphone app: Secondary (GPS) | Source stated not referenced | Temporally and Spatially | Measured, not  controlled for | Measured, not  controlled for | Not measured | Not measured | Not measured | Not measured | Not measured |
| Utilizing Crowdsourced Data for  Studies of Cycling and Air  Pollution Exposure: A Case Study Using Strava Data | Sun, Y. R.; Mobasheri, A. | 2017 | 2 | No | Europe (UK) | Cross-sectional | 365 days | 13,684 users | 12.40% | All Adults | App users | None | Yes (only final) | No | No metric | Yes (this is a  subsequent paper) | Previous paper(s) not  acknowledged | Yes, in this study | Yes | Smartphone GPS:  secondary | Smartphone app: Secondary (GPS) | Source fully references | Temporally and Spatially | Measured, not  controlled for | Not measured | Not measured | Not measured | Not measured | Not measured | Not measured |
| Where does bicycling for health happen? Analysing volunteered  geographic information through  place and plexus | Griffin, Greg P.; Jiao, Junfeng | 2015 | 1 | No | North America (USA) | Cross-sectional | 7 days | 16,278 trips | Unclear | All Adults | App users | None | Yes (only final) | No | Compared to  Population as a whole | No | NA | NA | Yes | Smartphone GPS:  secondary | Smartphone app: Secondary (GPS) | Source fully references | Temporally and Spatially | Measured, not  controlled for | Measured, not  controlled for | Not measured | Not measured | Not measured | Not measured | Not measured |
| Why do bicyclists take detours? A multilevel regression model using smartphone GPS data | Park Yujin; Akar Gulsah | 2019 | 1 | No | North America (USA) | Cross-sectional | NA | 73 | 20.50% | All Adults | NA | None | Yes (only final) | No | No metric | No | NA | NA | Yes | Smartphone GPS:  primary | Smartphone app: Primary (GPS) | Source fully references | NA | Measured, not  controlled for | Measured, not  controlled for | Not measured | Not measured | Not measured | Not measured | Not measured |
| Public Transit Use and Physical  Activity in Community-Dwelling  Older Adults: Combining GPS and Accelerometry to Assess | Voss Christine.; Sims Gould  Joanie.; Ashe Maureen C.;  McKay Heather A.; Pugh  Caitlin.; Winters Meghan. | 2016 | 1 | No | North America (Canada) | Cross-sectional | 7 days | 178 | 65% | Older Adults (65+) | NA | Meet health +address | Yes (only final) | No | Compared to excluded  Population | Yes (this is a  subsequent paper) | Results  summarise d | Yes, in referenced study or protocol  +in this study | No | GPS device +GIS | Accelerometer | Source nonexplicitly  mentioned | Temporally and Spatially | Measured, not  controlled for | Measured, not  controlled for | not  measured | Measured, not  controlled for | Measured, not  controlled for | Measured, not  controlled for | Measured, not  controlled for |
| Correspondence between objective and perceived walking times to urban destinations:  influence of physical activity,  neighbourhood walkability, and socio-demographics | Dewulf, B.; Neutens, T.;  Dyck, D. van;  Bourdeaudhuij, I. de; Weghe, N. van de | 2012 | 5 | Yes | Europe (Belgium) | Cross-sectional | 7 days | 1166 | 51.1 | Working  Population (18-65 years) | Live in area meeting specific  BE + SES criteria | Criteria required but not mentioned | Yes (only final) | No | No metric | Yes (this is a  subsequent paper) | Papers ref  but result not  summarise d | Yes, in referenced study or protocol  +in this study | No | GIS inferred | Accelerometer | Source fully references | Temporally and Spatially | Measured and  controlled for | Measured and  controlled for | Not measured | Measured and  controlled for | Measured and  controlled for | Not measured | Not measured |
| Neighborhood walkability and sedentary time in Belgian adults | Dyck, D. van; Cardon, G.;  Deforche, B.; Owen, N.;  Sallis, J. F.; Bourdeaudhuij,  I. de | 2010 | 5 | Yes | Europe (Belgium) | Cross-sectional | 7 days | 1166 | 52.1 | Working  Population (18-65 years) | Live in area meeting specific  BE + SES criteria | Meet PA criteria | Yes (only final) | No | Compared to  Population as a whole | Yes (first) | NA | Yes, in referenced study or protocol  +in this study | No | GIS inferred | Accelerometer | No data source info | Temporally and Spatially | Measured and  controlled for | Measured and  controlled for | Not measured | Measured and  controlled for | Measured and  controlled for | Not measured | Measured and  controlled for |
| Neighborhood SES and walkability are related to physical activity behavior in Belgian adults | Van Dyck, D.; Cardon, G.;  Deforche, B.; Sallis, J. F.;  Owen, N.; De  Bourdeaudhuij, I. | 2010 | 5 | Yes | Europe (Belgium) | Cross-sectional | 7 days | 1166 | 52.1 | Working  Population (18-65 years) | Live in area meeting specific  BE + SES criteria | Meet PA criteria | Yes (only final) | No | Compared to  Population as a whole | Yes (this is a  subsequent paper) | Previous paper(s) not  acknowledged | Yes, in this study | No | GIS inferred | Accelerometer | Source stated not referenced | Temporally and Spatially | Measured and  controlled for | Measured and  controlled for | Not measured | Measured and  controlled for | Measured and  controlled for | Not measured | Measured and  controlled for |
| Relationships between neighborhood walkability and adults' physical activity: How important is residential self-selection? | Van Dyck, D.; Cardon, G.;  Deforche, B.; Owen, N.; De Bourdeaudhuij, I. | 2011 | 5 | Yes | Europe (Belgium) | Cross-sectional | 7 days | 1166 | 51.9 | Working  Population (18-65 years) | Live in area meeting specific  BE + SES criteria | Criteria required but not mentioned | Partially described  (aggregated/ subsetted or unclear) | No | Compared to  Population as a whole | Yes (this is a  subsequent paper) | Papers ref  but result not  summarise d | Yes, in referenced study or protocol  +in this study | No | GIS inferred | Accelerometer | Source stated not referenced | Temporally and Spatially | Measured and  controlled for | Measured and  controlled for | Not measured | Measured and  controlled for | Measured and  controlled for | Not measured | Not measured |
| Relationship between neighborhood walkability and older adults' physical activity:  Results from the Belgian  Environmental Physical Activity  Study in Seniors (BEPAS Seniors) | Van Holle, V.; Van  Cauwenberg, J.; Van Dyck,  D.; Deforche, B.; Van de  Weghe, N.; De  Bourdeaudhuij, I. | 2014 | 5 | Yes | Europe (Belgium) | Cross-sectional | 7 days | 438 | 54.10% | Older Adults (65+) | Live in area meeting specific  BE + SES criteria | Meet PA criteria  + language criteria + address | Yes (only final) | No | No metric | Yes (this is a  subsequent paper) | Results  summarise d | Yes, in referenced study or protocol  +in this study | No | GIS inferred | Accelerometer | No data source info | Temporally and Spatially | Measured and  controlled for | Measured and  controlled for | Not measured | Measured and  controlled for | Measured and  controlled for | Measured and  controlled for | Not measured |
| Neighbourhood walkability, daily steps and utilitarian walking in Canadian adults | Hajna, S.; Ross, N. A.;  Joseph, L.; Harper, S.; Dasgupta, K. | 2015 | 2 | No | North America (Canada) | Cross-sectional | 7 days | 2949 | 51.40% | All Adults | NA | None | Yes (only final) | No | Representative recruitment method | Yes (first) | NA | NA | No | GIS inferred | Accelerometer | Source fully references | Temporally and Spatially | Measured and  controlled for | Measured and  controlled for | Not measured | Measured and  controlled for | Not measured | Measured and  controlled for | Not measured |
| Laboratory-Assessed Markers of  Cardiometabolic Health and  Associations with GIS-Based Measures of Active-Living Environments. | Hajna, Samantha;  Dasgupta, Kaberi; Ross, Nancy A | 2018 | 2 | No | North America (Canada) | Cross-sectional | 7 days | 2949 | 54.20% | All Adults | NA | Meet health criteria (including min distance) | Yes (only final) | No | Representative recruitment method | Yes (this is a  subsequent paper) | Results  summarise d | Yes, in referenced study or protocol  +in this study | No | GIS inferred | Accelerometer | Source fully references | Temporally and Spatially | Measured and  controlled for | Measured and  controlled for | Not measured | Measured and  controlled for | Measured and  controlled for | Measured and  controlled for | Measured and  controlled for |
| Activity space environment and dietary and physical activity behaviors: a pilot study | Zenk, S. N.; Schulz, A. J.;  Matthews, S. A.; Odoms-  Young, A.; Wilbur, J.;  Wegrzyn, L.; Gibbs, K.;  Braunschweig, C.; Stokes, C. | 2011 | 1 | No | North America (USA) | Pilot Study | 7 days | 97 | 75.00% | All Adults | NA | None | Yes (only final) | No | Compared to excluded  Population | No | NA | Yes, in referenced study or protocol  +in this study | No | GPS device +GIS | Accelerometer | Source stated not referenced | Temporally and Spatially | Measured and  controlled for | Measured and  controlled for | Measured and  controlled for | Measured and  controlled for | Measured and  controlled for | Not measured | Not measured |
| The association of the neighbourhood built environment with objectively measured  physical activity in older adults  with and without lower limb  osteoarthritis | Timmermans, E. J.; Schaap,  L. A.; Visser, M.; van der  Ploeg, H. P.; Wagtendonk,  A. J.; van der Pas, S.; Deeg,  D. J. | 2016 | 1 | No | Europe (Netherlands) | Case-control | 8 days | 206 | 46.10% | Older Adults (65+) | NA | None | Yes (only final) | Yes | Compared to excluded  Population | No | Na | NA | No | GPS device +GIS | Accelerometer | Source fully references | Temporally and Spatially | Measured and  controlled for | Measured and  controlled for | Not measured | Not measured | Measured and  controlled for | Measured and  controlled for | Not measured |
| The effect of moving to East  Village, the former London 2012  Olympic and Paralympic Games  Athletes' Village, on physical activity and adiposity (ENABLE  London): a cohort study | Nightingale, Claire M.;  Limb, Elizabeth S.; Ram,  Bina; Shankar, Aparna;  Clary, Christelle; Lewis,  Daniel; Cummins, Steven;  Procter, Duncan; Cooper,  Ashley R.; Page, Angie S.;  Ellaway, Anne  Giles-Corti, Billie  Whincup, Peter H.  Rudnicka, Alicja R.  Cook, Derek G.  Owen, Christopher G. | 2019 | 2 | No | Europe (UK) | Cohort,  Natural experiment | 7 days | 877 | 56.40% | All Adults | Live in area meeting specific  BE criteria | Meet BE criteria | Yes (only final) | No | Within sample comparison of included subgroups | Yes (first) | NA | Yes, in referenced study or protocol  +in this study | No | GIS inferred | Accelerometer | Source fully references | Temporally and Spatially | Measured and  controlled for | Measured and  controlled for | Measured and  controlled for | Measured, not  controlled for | Not measured | Not measured | Measured, not  controlled for |

| The effect of moving to East  Village, the former London 2012  Olympic and Paralympic Games  Athletes' Village, on mode of travel (ENABLE London study, a natural experiment). | Limb ES.; Procter DS.;  Cooper AR.; Page AS.;  Nightingale CM.; Ram B.;  Shankar A.; Clary C.; Lewis  D.; Cummins S.; Ellaway A.;  Giles-Corti B.; Whincup PH.; Rudnicka AR.; Cook DG.; Owen CG. | 2020 | 2 | No | Europe (UK) | Cohort,  Natural experiment | 7 days | 877 | 57.40% | All Adults | Live in area meeting specific  BE criteria | Meet BE criteria | Yes (only final) | No | Within sample comparison of included subgroups | Yes (this is a  subsequent paper) | Papers ref  but result not  summarise d | Yes, in referenced study or protocol  +in this study | No | GIS inferred | Accelerometer + GPS inferred | Source fully references | Temporally and Spatially | Measured and  controlled for | Measured and  controlled for | Measured and  controlled for | Measured, not  controlled for | Not measured | Not measured | Measured, not  controlled for |
| --- | --- | --- | --- | --- | --- | --- | --- | --- | --- | --- | --- | --- | --- | --- | --- | --- | --- | --- | --- | --- | --- | --- | --- | --- | --- | --- | --- | --- | --- | --- |
| Implications of the modifiable areal unit problem for assessing built environment correlates of  moderate and vigorous physical  activity | Houston, D. | 2014 | 2 | No | North America (USA) | Cross-sectional | 7 days | 55 | 71.00% | All Adults | NA | None | Yes (only final) | No | Compared to  Population as a whole | No | NA | NA | No | GPS device +GIS | Accelerometer | Source fully references | No aggregation | Measured and  controlled for | Measured and  controlled for | Measured and  controlled for | Measured and  controlled for | Not measured | Not measured | Measured and  controlled for |
| Bicycle boulevards and changes in physical activity and active  transportation: Findings from a natural experiment | Dill, J.; McNeil, N.; Broach, J.; Ma, L. | 2014 | 1 | No | North America (USA) | Natural Experiment | 5 days x 2  (baseline and follow-up) | 255 | 65% | All Adults | Parents | be able to cycle  and access bike | Yes(pre and post applying inclusion criteria) | No | No metric | No | NA | NA | No | GPS device +GIS | Accelerometer | Source fully references | Temporally | Measured and  controlled for | Measured and  controlled for | Not measured | Not measured | Measured and  controlled for | Measured and  controlled for | Measured and  controlled for |
| An assessment of the relevance of the home neighbourhood for  understanding environmental  influences on physical activity:  How far from home do people roam? | Hillsdon, M.; Coombes, E.; Griew, P.; Jones, A. | 2015 | 1 | No | Europe (UK) | Cross-sectional | 7 days | 195 | 57.90% | All Adults | Live in area meeting specific  BE + SES criteria | None | Yes (only final) | No | Representative recruitment method | No | NA | NA | No | GPS device +GIS | Accelerometer | NA | Temporally and Spatially | Measured, not  controlled  for, results  disaggregated by covariate | Measured, not  controlled  for, results  disaggregated by covariate | Not measured | Measured, not  controlled  for, results  disaggregated by covariate | Not measured | Not measured | Not measured |
| Physical Limitations, Walkability, Perceived Environmental  Facilitators and Physical Activity of Older Adults in Finland | Portegijs, E.; Keskinen, K. E.; Tsai, L. T.; Rantanen, T.; Rantakokko, M. | 2017 | 1 | No | Europe (Finland) | cross-  sectional | 7 days | 130 | 62.30% | Older Adults (65+) | Older Adults | Address criteria  (including community dwelling) | Yes (only final) | Yes | Within sample comparison of included subgroups | No | Na | NA | No | GIS inferred | Accelerometer | No data source info | Temporally and Spatially | Measured and  controlled for | Measured and  controlled for | Not measured | Not measured | Measured, not  controlled for | Not measured | Measured, not  controlled for |
| Transit Use, Physical Activity, and Body Mass Index Changes:  Objective Measures Associated  With Complete Street Light-Rail Construction | Brown, B. B.; Werner, C.  M.; Tribby, C. P.; Miller, H.  J.; Smith, K. R. | 2015 | 7 | No | North America (USA) | Quasi-experimental | 7 days | 536 | 51.00% | All Adults | Live in area meeting specific  BE criteria | Meet PA criteria  + language criteria | Yes (only final) | No | Within sample comparison of included subgroups | Yes (first) | NA | Yes, in this study | No | GPS device +GIS | Accelerometer | Source nonexplicitly  mentioned | Temporally and Spatially | Measured and  controlled for | Measured, not  controlled for | Measured and  controlled for | Not measured | Measured and  controlled for | Measured and  controlled for | Measured, not  controlled for |
| Environmental, behavioral, and psychological predictors of transit ridership: Evidence from a community intervention | Brown, Barbara B.; Werner,  Carol M.; Smith, Ken R.;  Tribby, Calvin P.; Miller,  Harvey J.; Jensen, Wyatt A.; Tharp, Doug | 2016 | 7 | No | North America (USA) | Quasi-experimental | 7 days | 536 | 51% | All Adults | Live in area meeting specific  BE criteria | Meet PA criteria  + language criteria | No participant characteristics described | No | Compared to  Population as a whole | Yes (this is a  subsequent paper) | Previous paper(s) not  acknowledged | Yes, in referenced study or protocol  +in this study | No | GPS device +GIS | Accelerometer | Source stated not referenced | Unclear | Measured, not  controlled for | Measured, not  controlled for | Measured, not  controlled for | Not measured | Not measured | Not measured | Not measured |
| Changes in bicycling over time associated with a new bike lane:  Relations with kilocalories energy expenditure and body mass index | Brown, Barbara B.; Tharp,  Douglas; Tribby, Calvin P.;  Smith, Ken R.; Miller,  Harvey J.; Werner, Carol M. | 2016 | 7 | No | North America (USA) | Quasi-experimental | 7 days | 536 | 51% | All Adults | Live in area meeting specific  BE criteria | Meet PA criteria  + language criteria | Only characteristics pre-applying  inclusion criteria/ subsection described | No | Within sample comparison of included subgroups | Yes (this is a  subsequent paper) | Previous paper(s) not  acknowledged | Yes, in referenced study or protocol  +in this study | No | GPS device +GIS | Accelerometer + GPS inferred | Source nonexplicitly  mentioned | Temporally and Spatially | Measured and  controlled for | Measured and  controlled for | Measured and  controlled for | Measured and  controlled for | Not measured | Not measured | Not measured |
| A Complete Street Intervention for  Walking to Transit, Non-transit  Walking, and Bicycling: A Quasi-  Experimental Demonstration of Increased Use | Brown, B. B.; Smith, K. R.;  Tharp, D.; Werner, C. M.;  Tribby, C. P.; Miller, H. J.; Jensen, W. | 2016 | 7 | No | North America (USA) | Quasi-experimental | 7 days | 536 | 51% | All Adults | Live in area meeting specific  BE criteria | Meet PA criteria  + language criteria | Yes (only final) | No | Within sample comparison of included subgroups | Yes (this is a  subsequent paper) | Previous paper(s) not  acknowledged | Yes, in referenced study or protocol  +in this study | No | GPS device +GIS | Accelerometer + GPS inferred | Source nonexplicitly  mentioned | Temporally and Spatially | Measured and  controlled for | Measured and  controlled for | Measured and  controlled for | Measured and  controlled for | Measured and  controlled for | Measured and  controlled for | Measured and  controlled for |
| Objectively measured active travel and uses of activity-friendly  neighborhood resources: Does  change in use relate to change in physical activity and BMI? | Brown, B. B.; Tharp, D.; Smith, K. R.; Jensen, W. A. | 2017 | 7 | No | North America (USA) | Quasi-experimental | 7 days | 536 | 51% | All Adults | Live in area meeting specific  BE criteria | Meet PA criteria  + language criteria | Only characteristics pre-applying  inclusion criteria/ subsection described | No | Compared to excluded  Population | Yes (this is a  subsequent paper) | Results  summarise d | Yes, in referenced study or protocol  +in this study | No | GPS device +GIS | Accelerometer | Source nonexplicitly  mentioned | Temporally and Spatially | Measured and  controlled for | Measured and  controlled for | Measured, not  controlled for | Not measured | Measured and  controlled for | Not measured | Measured and  controlled for |
| Sports facilities, shopping centers or homes: What locations are important for adults' physical activity? A cross-sectional study | Jansen, M.; Ettema, D.; Pierik, F.; Dijst, M. | 2016 | 3 | No | Europe (Netherlands) | Cross-sectional | 7 days | 308 | 54.90% | Working  Population (18-65 years) | Live in area meeting specific  BE criteria | Meet BE criteria | Yes (only final) | No | Compared to  Population as a whole | Yes (first) | NA | NA | No | GPS device +GIS | Accelerometer | Source fully references | Temporally | Measured and  controlled for | Measured and  controlled for | Measured and  controlled for | Measured and  controlled for | Measured and  controlled for | Measured and  controlled for | Measured and  controlled for |
| How do type and size of natural environments relate to physical activity behavior? | Jansen, F.; Ettema, D.;  Kamphuis, C.; Pierik, F.; Dijst, M. | 2017 | 3 | No | Europe (Netherlands) | Cross-sectional | 7 days | 308 | 54.10% | Working  Population (18-65 years) | Live in area meeting specific  BE criteria | Meet BE criteria | Yes (only final) | No | Compared to  Population as a whole | Yes (this is a  subsequent paper) | Results  summarise d | Yes, in referenced study or protocol  +in this study | No | GPS device +GIS | Accelerometer + GPS inferred | Source fully references | Temporally | Measured and  controlled for | Measured and  controlled for | Measured and  controlled for | Not measured | Measured and  controlled for | Not measured | Measured and  controlled for |
| Neighborhood-based PA and its environmental correlates: a GIS-  and GPS based cross-sectional study in the Netherlands | Jansen, M.; Kamphuis, C. B. M.; Pierik, F. H.; Ettema, D.  F.; Dijst, M. J. | 2018 | 3 | No | Europe (Netherlands) | Cross-sectional | 7 days | 308 | 54.90% | Working  Population (18-65 years) | Live in area meeting specific  BE criteria | Meet BE criteria | Yes (only final) | No | Compared to  Population as a whole | Yes (this is a  subsequent paper) | Papers ref  but result not  summarise d | Yes, in referenced  study or protocol | No | GPS device +GIS | Accelerometer | Source fully references | Temporally and Spatially | Measured and  controlled for | Measured and  controlled for | Measured and  controlled for | Not measured | Measured and  controlled for | Not measured | Measured and  controlled for |
| One size doesn't fit all: Cross-sectional associations between  neighborhood walkability, crime and physical activity depends on age and sex of residents | Richardson, A. S.; Troxel,  W. M.; Ghosh-Dastidar, M.  B.; Beckman, R.; Hunter, G.  P.; DeSantis, A. S.;  Colabianchi, N.; Dubowitz, T. | 2017 | 1 | No | North America (USA) | Cross-sectional | 7 days | 791 | 77.11% | All Adults | Specific ethnicity/ nationality +SES criteria | None | Yes (only final) | No | Representative recruitment method | No | Na | Yes, in referenced study or protocol  +in this study | No | GIS inferred | Accelerometer | Source fully references | Temporally and Spatially | Measured and  controlled for | Measured and  controlled for | Measured, not  controlled for | Measured and  controlled for | Measured and  controlled for | Measured and  controlled for | Not measured |
| Living Close to Natural Outdoor  Environments in Four European  Cities: Adults' Contact with the  Environments and Physical Activity | Triguero-Mas, M.; Donaire-  Gonzalez, D.; Seto, E.;  Valentin, A.; Smith, G.;  Martinez, D.; Carrasco-  Turigas, G.; Masterson, D.; van den Berg, M.; Ambros,  A.; Martinez-Iniguez, T.;  Dedele, A.; Hurst, G.; Ellis, N.; Grazulevicius, T.;  Voorsmit, M.; Cirach, M.;  Cirac-Claveras, J.; Swart,  W.; Clasquin, E.; Maas, J.;  Wendel-Vos, W.; Jerrett,  M.; Grazuleviciene, R.;  Kruize, H.; Gidlow, C. J.; Nieuwenhuijsen, M. J. | 2017 | 1 | No | Europe (UK, Spain,  The Netherlands, Lithuania) | Cross-sectional | 7 days | 408 | 53.68% | All Adults | NA | Meet PA criteria | Yes (only final) | No | No metric | No | NA | Yes, in referenced study or protocol  +in this study | Yes | Smartphone GPS:  primary | Smartphone app: Primary  (accelerometer/gyroscope) | Source fully references | Temporally and Spatially | Measured and  controlled for | Measured and  controlled for | Not measured | Measured and  controlled for | Measured and  controlled for | Not measured | Not measured |

| Walk Score, Transportation Mode  Choice, and Walking Among French Adults: A GPS,  Accelerometer, and Mobility Survey Study | Duncan, D. T.; Meline, J.;  Kestens, Y.; Day, K.; Elbel, B.; Trasande, L.; Chaix, B. | 2016 | 2 | No | Europe (France) | Cross-sectional and Case crossover | 7 days | 234 | Not reported | All Adults | NA | None | No participant characteristics described | No | Compared to excluded  Population | Yes (first) | NA | Yes, in referenced study or protocol  +in this study | No | GPS device +GIS | Accelerometer | No data source info | Temporally and Spatially | Not measured | Measured and  controlled for | Not measured | Measured and  controlled for | Measured and  controlled for | Measured and  controlled for | Measured and  controlled for |
| --- | --- | --- | --- | --- | --- | --- | --- | --- | --- | --- | --- | --- | --- | --- | --- | --- | --- | --- | --- | --- | --- | --- | --- | --- | --- | --- | --- | --- | --- | --- |
| A GPS-Based Methodology to  Analyze Environment-Health  Associations at the Trip Level:  Case-Crossover Analyses of Built Environments and Walking | Chaix, B.; Kestens, Y.;  Duncan, D. T.; Brondeel, R.; Meline, J.; El Aarbaoui, T.; Pannier, B.; Merlo, J. | 2016 | 2 | No | Europe (France) | Cross-sectional and Case crossover | 7 days | 234 | 34.90% | All Adults | NA | None | Yes(pre and post applying inclusion criteria) | No | No metric | Yes (this is a  subsequent paper) | Previous paper(s) not  acknowledged | Yes, in referenced study or protocol  +in this study | No | GPS device +GIS | Accelerometer | Source fully references | Temporally and Spatially | Measured and  controlled for | Not measured | Not measured | Measured and  controlled for | Measured and  controlled for | Measured and  controlled for | Measured and  controlled for |
| Neighborhood Environment and  Psychosocial Correlates of Adults’ Physical Activity | BRIAN E. SAELENS1, JAMES F. SALLIS2, LAWRENCE D.  FRANK3,4, KELLI L. CAIN2,  TERRY L. CONWAY2, JAMES E. CHAPMAN4, DONALD J.  SLYMEN4, and JACQUELINE KERR2 | 2012 | 4 | Yes | North America (USA) | Cross-sectional | 7 days | 2199 | 47.9%% | Working  Population (18-65 years) | Live in area meeting specific  BE + SES criteria | Meet health + PA  + language criteria + address | Yes (only final) | No | Compared to  Population as a whole | Yes (this is a  subsequent paper) | Results  summarise d | Yes, in referenced study or protocol  +in this study | No | GIS inferred | Accelerometer | Source nonexplicitly  mentioned | Temporally and Spatially | Measured and  controlled for | Measured and  controlled for | Measured and  controlled for | Measured and  controlled for | Measured and  controlled for | Measured and  controlled for | Measured and  controlled for |
| Is Your Neighborhood Designed to  Support Physical Activity? A Brief Streetscape Audit Tool | Sallis, J. F.; Cain, K. L.;  Conway, T. L.; Gavand, K.  A.; Millstein, R. A.;  Geremia, C. M.; Frank, L. D.; Saelens, B. E.; Glanz, K.; King, A. C. | 2015 | 4 | Yes | North America (USA) | Cross-sectional | 7 days | 2199 | 48.20% | Working  Population (18-65 years) | Live in area meeting specific  BE + SES criteria | None | Yes (only final) | No | Compared to excluded  Population | Yes (first) | NA | Na | No | GIS inferred | Accelerometer | No data source info | Temporally and Spatially | Measured and  controlled for | Measured and  controlled for | Measured and  controlled for | Measured and  controlled for | Measured and  controlled for | Measured and  controlled for | Not measured |
| Patterns of Walkability, Transit, and Recreation Environment for  Physical Activity | Adams, M. A.; Todd, M.;  Kurka, J.; Conway, T. L.; Cain, K. L.; Frank, L. D.; Sallis, J. F. | 2015 | 4 | Yes | North America (USA) | Cross-sectional | 7 days | 2199 | 48.20% | Working  Population (18-65 years) | Live in area meeting specific  BE + SES criteria | None | Yes (only final) | No | Within sample comparison of included subgroups | Yes (this is a  subsequent paper) | Previous paper(s) not  acknowledged | Yes, in this study | No | GIS inferred | Accelerometer | No data source info | Temporally and Spatially | Measured and  controlled for | Measured and  controlled for | Measured and  controlled for | Measured and  controlled for | Measured and  controlled for | Measured and  controlled for | Not measured |
| Is the relationship between the built environment and physical  activity moderated by perceptions of crime and safety? | Bracy, N. L.; Millstein, R. A.;  Carlson, J. A.; Conway, T. L.; Sallis, J. F.; Saelens, B. E.;  Kerr, J.; Cain, K. L.; Frank, L. D.; King, A. C. | 2014 | 4,4 | Yes | North America (USA) | Cross-sectional | 7 days | 2199 | 49.04 | All Adults | Live in area meeting specific  BE + SES criteria | Meet PA criteria  + language criteria + address | Yes (only final) | No | No metric | Yes (first) | NA | NA | No | GIS inferred | Accelerometer | Source nonexplicitly  mentioned | Temporally and Spatially | Measured and  controlled for | Measured and  controlled for | Measured and  controlled for | Not measured | Measured and  controlled for | Measured and  controlled for | Not measured |
| Neighborhood environment and physical activity among older  adults: do the relationships differ by driving status? | Ding, D.; Sallis, J. F.;  Norman, G. J.; Frank, L. D.; Saelens, B. E.; Kerr, J.;  Conway, T. L.; Cain, K.;  Hovell, M. F.; Hofstetter, C. R.; King, A. C. | 2014 | 4 | Yes | North America (USA) | Cross-sectional | 7 days | 861 | 56% | Older Adults (65+) | Live in area meeting specific  BE + SES criteria | Meet PA criteria  + language criteria + address | Yes (only final) | No | No metric | Yes (this is a  subsequent paper) | Na | Yes, in referenced study or protocol  +in this study | No | GIS inferred | Accelerometer | No data source info | Temporally and Spatially | Measured and  controlled for | Measured and  controlled for | Measured and  controlled for | Not measured | Measured and  controlled for | Measured and  controlled for | Not measured |
| Physical Activity in Older Adults:  an Ecological Approach | Thornton, C. M.; Kerr, J.; Conway, T. L.; Saelens, B.  E.; Sallis, J. F.; Ahn, D. K.;  Frank, L. D.; Cain, K. L.; King, A. C. | 2017 | 4 | No | North America (USA) | Cross-sectional | 7 days | 861 | 53.10% | Older Adults (65+) | Live in area meeting specific  BE + SES criteria | Meet PA criteria  + language criteria | Yes (only final) | No | Compared to  Population as a whole | Yes (this is a  subsequent paper) | Results  summarise d | Yes, in referenced study or protocol  +in this study | No | GIS inferred | Accelerometer | Source nonexplicitly  mentioned | Temporally and Spatially | Measured and  controlled for | Measured and  controlled for | Measured and  controlled for | Measured and  controlled for | Measured and  controlled for | Measured and  controlled for | Not measured |
| GIS-measured walkability, transit, and recreation environments in  relation to older Adults' physical activity: A latent profile analysis | Todd, M.; Adams, M. A.;  Kurka, J.; Conway, T. L.;  Cain, K. L.; Buman, M. P.; Frank, L. D.; Sallis, J. F.; King, A. C. | 2016 | 4 | no | North America (USA) | Cross-sectional | 7 days | 861 | 52.10% | Older Adults (65+) | Live in area meeting specific  BE + SES criteria | None | Yes(pre and post applying inclusion criteria) | No | Representative recruitment method | Yes (this is a  subsequent paper) | Previous studies use of data  acknowledged but not results  reported or referenced | Yes, in referenced study or protocol  +in this study | No | GIS inferred | Accelerometer | Source nonexplicitly  mentioned | Temporally and Spatially | Measured and  controlled for | Measured and  controlled for | Measured and  controlled for | Measured and  controlled for | Measured and  controlled for | Measured and  controlled for | Measured and  controlled for |
| Linking objectively measured physical activity with objectively  measured urban form: findings  from SMARTRAQ | Frank, L. D.; Schmid, T. L.; Sallis, J. F.; Chapman, J.; Saelens, B. E. | 2005 | 1 | No | North America (USA) | Cross-sectional | 2 days | 357 | 55.70% | Working  Population (18-65 years) | Live in area meeting specific  BE criteria | None | Yes (only final) | No | Compared to  Population as a whole | No | NA | NA | No | GIS inferred | Accelerometer | Source stated not referenced | Temporally and Spatially | Measured and  controlled for | Measured and  controlled for | Measured and  controlled for | Not measured | Measured and  controlled for | Not measured | Not measured |
| Walkability parameters, active transportation and objective  physical activity: moderating and mediating effects of motor  vehicle ownership in a cross-  sectional study | Eriksson, U.; Arvidsson, D.; Gebel, K.; Ohlsson, H.; Sundquist, K. | 2012 | 2 | No | Europe (Sweden) | Cross-sectional | 7 days | 2269 | 55% | Working  Population (18-65 years) | Live in area meeting specific  BE + SES criteria | Meet PA criteria  + language criteria + address | Yes (only final) | No | No metric | Yes (this is a  subsequent paper) | Results  summarise d | Yes, in referenced study or protocol  +in this study | No | GIS inferred | Accelerometer | Source stated not referenced | Temporally and Spatially | Measured and  controlled for | Measured and  controlled for | Not measured | Measured and  controlled for | Not measured | Measured and  controlled for | Not measured |
| Neighborhood walkability, physical activity, and walking behavior: The Swedish  Neighborhood and Physical Activity (SNAP) study | Sundquist Kristina.; Eriksson Ulf.; Kawakami Naomi.;  Skog Lars.; Ohlsson Henrik.; Arvidsson Daniel | 2011 | 2 | No | Europe (Sweden) | Cross-sectional | 7 days | 2269 | 55% | Working  Population (18-65 years) | Live in area meeting specific  BE + SES criteria | Meet PA criteria  + language criteria + address | Yes (only final) | No | Compared to excluded  Population | Yes (first) | NA | NA | No | GIS inferred | Accelerometer | Source stated not referenced | Temporally and Spatially | Measured and  controlled for | Measured and  controlled for | Not measured | Measured and  controlled for | Not measured | Measured and  controlled for | Not measured |
| The relationship between  Mediterranean built environment and outdoor physical activity: evidence from GPS and  accelerometer data among young  adults in Barcelona | Delcl√≤s-Ali√≥, X.; Vich, G.; Miralles-Guasch, C. | 2020 | 1 | No | Europe (Spain) | Cross-sectional | 7 days | 138 | 52.90% | All Adults | NA | None | Yes (only final) | No | No metric | No | NA | NA | No | GPS device +GIS | Accelerometer | No data source info | No aggregation | Measured, not  controlled for | Measured, not  controlled for | Not measured | Not measured | Not measured | Not measured | Measured, not  controlled for |
| Temperature and Rain Moderate the Effect of Neighborhood  Walkability on Walking Time for Seniors in Barcelona. | Delcl√≤s-Ali√≥ X.; Marquet O.; Vich G.; Schipperijn J.;  Zhang K.; Maciejewska M.; Miralles-Guasch C. | 2019 | 2 | No | Europe (Spain) | Cross-sectional | 7 days | 227 | 56% | Older Adults (65+) | Older Adults | Meet health criteria (including min distance) | Partially described  (aggregated/ subsetted or unclear) | No | No metric | Yes (first) | Papers ref  but result not  summarise d | Yes, in referenced study or protocol  +in this study | No | GPS device +GIS | Accelerometer + GPS inferred | No data source info | Temporally and Spatially | Measured and  controlled for | Measured and  controlled for | Not measured | Not measured | Not measured | Not measured | Not measured |
| Natural Landscape, Infrastructure, and Health: The Physical Activity  Implications of Urban Green  Space Composition among the Elderly. | Miralles-Guasch, Carme;  Dopico, Javier; Delclos-Alio,  Xavier; Knobel, Pablo;  Marquet, Oriol; ManejaZaragoza, Roser;  Schipperijn, Jasper; Vich, Guillem | 2019 | 2 | No | Europe (Spain) | Cross-sectional | 7 days | 227 | 44.40% | Older Adults (65+) | Older Adults | None | Yes (only final) | No | No metric | Yes (this is a  subsequent paper) | Previous paper(s) not  acknowledged | Yes, in this study | No | GPS device +GIS | Accelerometer | Source fully references | Temporally | Measured and  controlled for | Measured and  controlled for | Not measured | Not measured | Not measured | Not measured | Not measured |
| Comparisons of Physical Activity and Walking Between Korean  Immigrant and White Women in King County, WA | Baek, S. R.; Moudon, A. V.; Saelens, B. E.; Kang, B.; Hurvitz, P. M.; Bae, C. C. | 2016 | 1,9 | No | North America (USA) | Case-control | 7 days | 69 | 100% | Working  Population (18-65 years) | Women +  Specific ethnicity/ nationality | Meet health + ethnicity + address criteria | Yes (only final) | No | Within sample comparison of included subgroups | Yes (this is a  subsequent paper) | Papers ref  but result not  summarise d | Yes, in referenced study or protocol  +in this study | No | GPS device +GIS | Accelerometer + GPS inferred | Source fully references | Temporally and Spatially | Measured and  controlled for | Measured and  controlled for | Measured and  controlled for | Measured and  controlled for | Not measured | Not measured | Measured and  controlled for |
| Light Rail Leads to More Walking Around Station Areas | Huang, R.; Moudon, A. V.; Zhou, C.; Stewart, O. T.; Saelens, B. E. | 2017 | 9 | No | North America (USA) | Cross-sectional and Case crossover | 7 days ,7 days x2  (baseline and follow-up) | 675 | 63.00% | All Adults | Live in area meeting specific  BE criteria | Meet PA criteria  + language criteria + address | Yes (only final) | No | Compared to excluded  Population | Yes (first) | Papers ref  but result not  summarise d | Yes, in this study | No | GPS device +GIS | Accelerometer + GPS inferred | No data source info | Temporally and Spatially | Measured and  controlled for | Measured and  controlled for | Measured and  controlled for | Measured and  controlled for | Measured and  controlled for | Not measured | Not measured |

| Higher residential and employment densities are  associated with more objectively  measured walking in the home  neighborhood | Huang, R.; Moudon, A.V.; Zhou, C.; Saelens, B.E. | 2019 | 9 | No | North America (USA) | Cross-sectional and Case crossover | 7 days ,7 days x2  (baseline and follow-up) | 676 | 63.10% | All Adults | Live in area meeting specific  BE criteria | Meet PA criteria  + language criteria + address | Yes (only final) | No | No metric | Yes (this is a  subsequent paper) | Results  summarise d | Yes, in referenced study or protocol  +in this study | No | GPS device +GIS | Accelerometer + GPS inferred | Source stated not referenced | Temporally and Spatially | Measured and  controlled for | Measured and  controlled for | Measured and  controlled for | Measured, not  controlled for | Measured and  controlled for | Not measured | Not measured |
| --- | --- | --- | --- | --- | --- | --- | --- | --- | --- | --- | --- | --- | --- | --- | --- | --- | --- | --- | --- | --- | --- | --- | --- | --- | --- | --- | --- | --- | --- | --- |
| Differences in behavior, time, location, and built environment between objectively measured utilitarian and recreational walking | Kang, B.; Moudon, A. V.; Hurvitz, P. M.; Saelens, B. E. | 2017 | 9 | No | North America (USA) | Cross-sectional and Case crossover | 7 days ,7 days x2  (baseline and follow-up) | 677 | 62.40% | All Adults | Live in area meeting specific  BE criteria | None | Yes (only final) | No | No metric | Yes (this is a  subsequent paper) | Previous paper(s) not  acknowledged | Yes, in this study | No | GPS device +GIS | Accelerometer + GPS inferred | Source stated not referenced | Temporally and Spatially | Measured, not  controlled for | Measured, not  controlled for | Measured, not  controlled for | Measured, not  controlled for | Measured, not  controlled for | Not measured | Measured, not  controlled for |
| The association between park visitation and physical activity  measured with accelerometer,  GPS, and travel diary | Stewart, O. T.; Moudon, A. V.; Fesinmeyer, M. D.;  Zhou, C.; Saelens, B. E. | 2016 | 9 | No | North America (USA) | Cross-sectional and Case crossover | 7 days ,7 days x2  (baseline and follow-up) | 678 | 63.40% | All Adults | Live in area meeting specific  BE criteria | Meet PA criteria  + language criteria + address | Yes (only final) | No | Within sample comparison of included subgroups | Yes (this is a  subsequent paper) | Papers ref  but result not  summarise d | Yes, in this study | No | GPS device +GIS | Accelerometer + GPS inferred | Source stated not referenced | Temporally and Spatially | Measured and  controlled for | Measured and  controlled for | Measured and  controlled for | Measured and  controlled for | Measured and  controlled for | Not measured | Not measured |
| The association between park facilities and the occurrence of physical activity during park visits | Stewart, O. T.; Moudon, A. V.; Littman, A.; Seto, E.; Saelens, B. E. | 2018 | 9 | No | North America (USA) | Cross-sectional and Case crossover | 7 days ,7 days x2  (baseline and follow-up) | 679 | 62.70% | All Adults | Live in area meeting specific  BE criteria | Meet PA criteria  + language criteria + address | Yes (only final) | No | Within sample comparison of included subgroups | Yes (this is a  subsequent paper) | Papers ref  but result not  summarise d | Yes, in referenced study or protocol  +in this study | No | GPS device +GIS | Accelerometer + GPS inferred | Source nonexplicitly  mentioned | Temporally and Spatially | Measured and  controlled for | Measured and  controlled for | Measured and  controlled for | Measured and  controlled for | Measured and  controlled for | Not measured | Not measured |
| The Association Between Park  Facilities and Duration of Physical Activity During Active Park Visits. | Stewart, Orion T; Moudon,  Anne Vernez; Littman,  Alyson J; Seto, Edmund; Saelens, Brian E | 2018 | 9 | No | North America (USA) | Cross-sectional and Case crossover | 7 days ,7 days x2  (baseline and follow-up) | 680 | 64% | All Adults | Live in area meeting specific  BE criteria | Meet PA criteria  + language criteria + address | Yes (only final) | No | No metric | Yes (this is a  subsequent paper) | Results  summarise d | Yes, in referenced study or protocol  +in this study | No | GPS device +GIS | Accelerometer + GPS inferred | Source nonexplicitly  mentioned | Temporally and Spatially | Measured, not  controlled for | Measured, not  controlled for | Measured, not  controlled for | Measured, not  controlled for | Measured, not  controlled for | Not measured | Not measured |
| Why neighborhood park proximity is not associated with total  physical activity | Stewart, O. T.; Moudon, A. V.; Littman, A. J.; Seto, E.; Saelens, B. E. | 2018 | 9 | No | North America (USA) | Cross-sectional and Case crossover | 7 days ,7 days x2  (baseline and follow-up) | 681 | 63% | All Adults | Live in area meeting specific  BE criteria | Meet PA criteria  + language criteria + address | Yes (only final) | No | Within sample comparison of included subgroups | Yes (this is a  subsequent paper) | Papers ref  but result not  summarise d | Yes, in referenced study or protocol  +in this study | No | GPS device +GIS | Accelerometer + GPS inferred | Source nonexplicitly  mentioned | Temporally and Spatially | Measured and  controlled for | Measured and  controlled for | Measured and  controlled for | Measured and  controlled for | Measured and  controlled for | Not measured | Not measured |
| Comparing Associations Between the Built Environment and  Walking in Rural Small Towns and a Large Metropolitan Area | Stewart, O. T.; Moudon, A.  V.; Saelens, B. E.; Lee, C.;  Kang, B. J.; Doescher, M. P. | 2016 | 1,9 | No | North America (USA) | Cross-sectional | 7 days | 324 | 65.50% | All Adults | Live in area meeting specific  BE criteria | Meet PA criteria  + language criteria + address | Yes (only final) | No | Within sample comparison of included subgroups | Yes (this is a  subsequent paper) | Papers ref  but result not  summarise d | Yes, in referenced study or protocol  +in this study | No | GPS device +GIS | Accelerometer + GPS inferred | No data source info | Temporally and Spatially | Measured and  controlled for | Measured and  controlled for | Measured and  controlled for | Measured and  controlled for | Measured and  controlled for | Measured and  controlled for | Not measured |
| Characteristics of the environment and physical activity in midlife:  Findings from UK Biobank | Smith, Lindsey; Panter, Jenna; Ogilvie, David | 2019 | 1 | No | Europe (UK) | Cross-sectional | 7 days | 65,967 | 54.40% | All Adults | NA | Address criteria  (including community dwelling) | Yes(pre and post applying inclusion criteria) | No | Compared to  Population as a whole | No | NA | Yes, in referenced study or protocol  +in this study | No | GIS inferred | Accelerometer | Source fully references | Temporally and Spatially | Measured and  controlled for | Measured and  controlled for | Measured and  controlled for | Measured and  controlled for | Measured and  controlled for | Not measured | Measured and  controlled for |
| What are the associations between neighbourhood  walkability and sedentary time in  New Zealand adults? the URBAN cross-sectional study | Hinckson, E.; Cerin, E.;  Mavoa, S.; Smith, M.;  Badland, H.; Witten, K.;  Kearns, R.; Schofield, G. | 2017 | 2 | Yes | Oceania (New Zealand) | Cross-sectional | 7 days | 1989 | 57.90% | Working  Population (18-65 years) | Live in area meeting specific  BE criteria + ethnicity | Meet PA criteria  + language criteria + address | Yes (only final) | No | Compared to excluded  Population | Yes (first) | NA | NA | No | GIS inferred | Accelerometer | Source stated not referenced | Temporally and Spatially | Measured and  controlled for | Measured and  controlled for | Measured and  controlled for | Measured and  controlled for | Measured and  controlled for | Measured and  controlled for | Measured and  controlled for |
| How Do Neighbourhood  Definitions Influence the  Associations between Built Environment and Physical Activity?. | Mavoa, Suzanne; Bagheri,  Nasser; Koohsari,  Mohammad J; Kaczynski,  Andrew T; Lamb, Karen E;  Oka, Koichiro; O'Sullivan, David; Witten, Karen | 2019 | 2 | Yes | Oceania (New Zealand) | Cross-sectional | 7 days | 1989 | Not reported | Not reported | Live in area meeting specific  BE + SES criteria | None | No participant characteristics described | No | No metric | Yes (this is a  subsequent paper) | Papers ref  but result not  summarise d | Yes, in referenced study or protocol  +in this study | No | GIS inferred | Accelerometer | Source stated not referenced | Temporally and Spatially | Measured and  controlled for | Measured and  controlled for | Measured and  controlled for | Measured and  controlled for | Measured and  controlled for | Measured and  controlled for | Measured and  controlled for |
| Cross Sectional Association between Spatially Measured  Walking Bouts and Neighborhood Walkability | Hwang, L. D.; Hurvitz, P. M.; Duncan, G. E. | 2016 | 1 | No | North America (USA) | Cross-sectional | 14 days | 106 | 75.50% | All Adults | Twins | None | Yes (only final) | No | Compared to  Population as a whole | No | Na | NA | No | GPS device +GIS | Accelerometer + GPS inferred | No data source info | Temporally and Spatially | Measured and  controlled for | Measured and  controlled for | Not measured | Measured and  controlled for | Not measured | Not measured | Not measured |
| Destinations That Older Adults  Experience Within Their GPS  Activity Spaces Relation to  Objectively Measured Physical Activity | Hirsch, J. A.; Winters, M.; Ashe, M. C.; Clarke, P.; McKay, H. | 2016 | 1 | No | North America (Canada) | Cross-sectional | 7 days | 77 | 66.20% | Older Adults (65+) | Low income | Meet PA criteria  + language criteria | Yes (only final) | No | No metric | No | NA | NA | No | GPS device +GIS | Accelerometer | Source fully references | Temporally and Spatially | Measured and  controlled for | Measured and  controlled for | Not measured | Measured, not  controlled for | Measured, not  controlled for | Measured, not  controlled for | Not measured |
| The influence of the local neighbourhood environment on  walking levels during the walking for wellbeing in the west  pedometer-based community  intervention | Robertson, L. B.; Ward  Thompson, C.; Aspinall, P.;  Millington, C.; McAdam, C.; Mutrie, N. | 2012 | 1 | No | Europe (UK) | Randomise d-control trial | 7 days x  4(Baseline,  3 months,  6 months,  12 months) | 76 | 71.10% | Working  Population (18-65 years) | NA | Meet PA criteria | Yes(pre and post applying inclusion criteria) | No | Compared to excluded  Population | No | NA | NA | No | GIS inferred | Pedometer | Source stated not referenced | Temporally and Spatially | Measured and  controlled for | Measured and  controlled for | Not measured | Measured and  controlled for | Not measured | Not measured | Not measured |
