# Supplementary material for: Objectively measuring the association between the built environment and physical activity: a systematic review and reporting framework
Source: Int J Behav Nutr Phys Act. 2022 Sep 14;19:119. doi: 10.1186/s12966-022-01352-7 (PMC9476279; doi:10.1186/s12966-022-01352-7)

Additional file C: Associations between physical activity (include cut-points) and the built environment metrics across all included studies (N=94). The number in the cell indicates number of studies investigating the association and the cell colour indicates the overall association between the metrics across all included studies (1.0 BE promotes PA to -1.0 BE impedes PA)

Page39of40

Page40of40


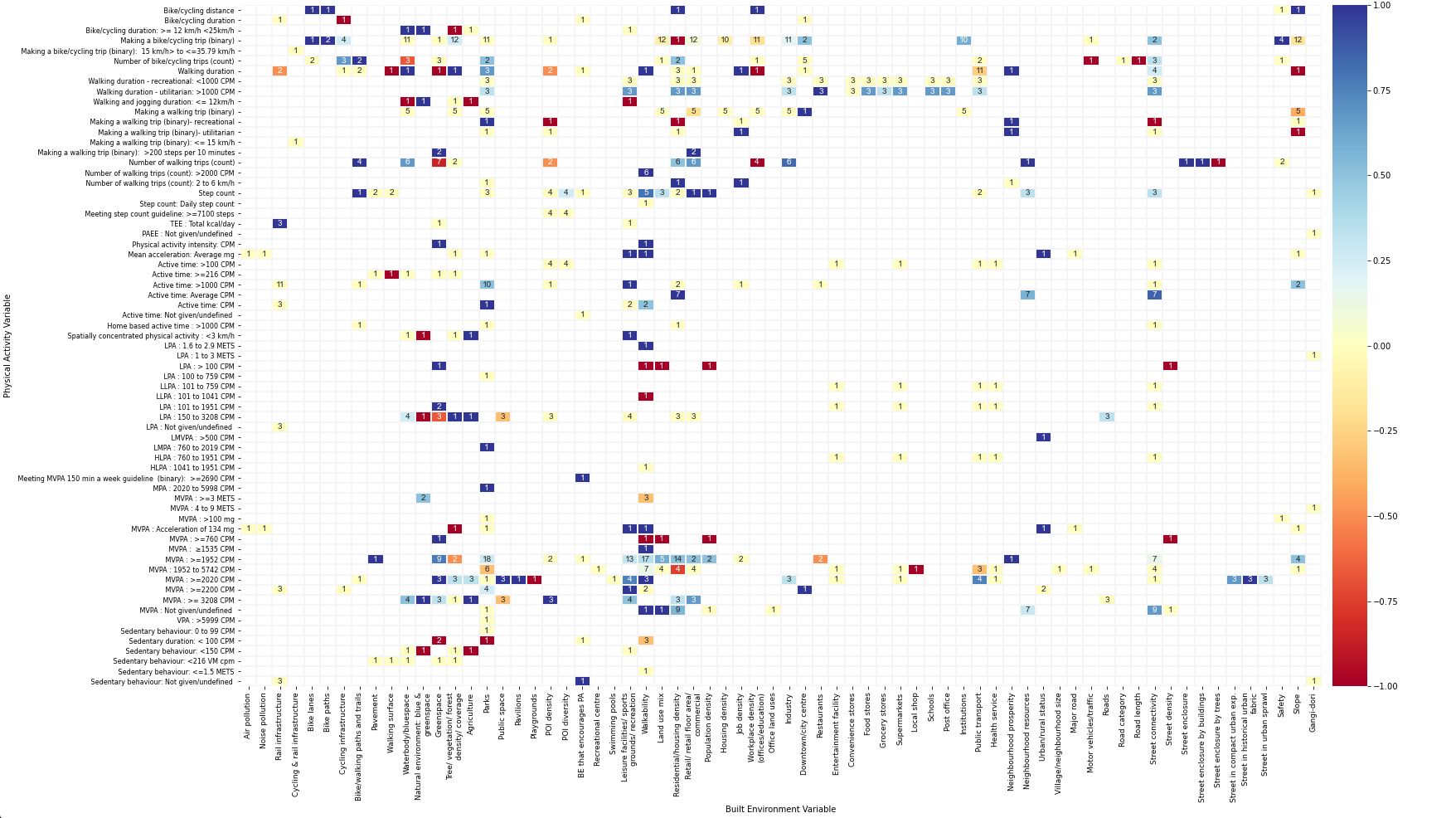

Supplement: Supplementary file 3 — Additional file 3. Associations between physical activity (include cut-points) and the built environment metrics across all included studies (N=94). The number in the cell indicates number of studies investigating the association and the cell colour indicates the overall association between the metrics across all included studies (1.0 BE promotes PA to -1.0 BE impedes PA). [file 12966_2022_1352_MOESM3_ESM.docx]
